# From bottleneck to boom: Polyploidy, genetic instability and response to artificial selection resolve the peanut paradox

**DOI:** 10.1111/tpj.70618

**Published:** 2025-12-24

**Authors:** Samuele Lamon, Brian L. Abernathy, Soraya C. M. Leal‐Bertioli, David J. Bertioli

**Affiliations:** ^1^ Institute of Plant Breeding, Genetics and Genomics University of Georgia Athens Georgia 30602 USA; ^2^ Center for Applied Genetic Technologies University of Georgia Athens Georgia 30602 USA; ^3^ Department of Plant Pathology University of Georgia Athens Georgia 30602 USA; ^4^ Department of Crop & Soil Sciences University of Georgia Athens Georgia 30602 USA

**Keywords:** *Arachis hypogaea*, polyploidy, neoallotetraploid, domestication, phenotypic diversity, artificial selection, homoeologous exchange, genome instability, crop evolution, *Arachis duranensis*, *Arachis ipaënsis*

## Abstract

This study, the second in a three‐part series, shows how peanut's polyploid origin enabled rapid diversification and enhanced domestication potential. Building on the knowledge that cultivated peanut (*Arachis hypogaea*) originated from a narrow hybridization between *Arachis duranensis* and *Arachis ipaënsis* less than 10 000 years ago, we are confronted with a paradox: how did such a narrow origin give rise to so much diversity—two subspecies, six botanical varieties, and thousands of landraces differing in growth habit, seed size, and pod morphology? Although several diploid *Arachis* species were cultivated earlier, only the allotetraploid became fully domesticated and widely adopted. The global success of peanut, despite its narrow genetic origin, suggests that polyploidization itself facilitated domestication. To test this hypothesis, we investigated how the two diploid progenitors and neoallotetraploids derived from a single hybridization and polyploidization event responded under artificial selection. In a pollinator‐free greenhouse, we advanced lineages of the neoallotetraploid and its diploid parents over 6 years, selecting for divergent seed weights. The neoallotetraploid showed a much stronger response to artificial selection than its diploid parents, while also spontaneously generating diverse phenotypic variation—including flower color, pod reticulation, and chlorophyll content—traits that distinguish *A. hypogaea* subspecies and landraces. These traits mirrored directional shifts in parental genome dosage caused by homoeologous exchange, supporting a causal connection with phenotype. These findings offer a compelling rationale for a domestication advantage in polyploid peanut, and provide a living demonstration of how a single ancestral tetraploid, despite an extreme genetic bottleneck, generates a phenotypic boom.

## Significance Statement

Cultivated peanut presents a paradox: despite an extremely narrow and relatively recent genetic origin, it displays remarkable phenotypic diversity. We show that a single neoallotetraploid lineage—a close reproduction of the ancestral peanut—spontaneously generates heritable phenotypic variation and responds strongly to artificial selection. This resolves the paradox and provides strong evidence for a polyploid advantage for domestication, consistent with the historical pattern in which only the polyploid lineage, *Arachis hypogaea*, became fully domesticated.

## INTRODUCTION

Cultivated peanut (*Arachis hypogaea*) originated less than 10 000 years ago from a hybridization event between two diploid wild species, *Arachis duranensis* and *Arachis ipaënsis*; likely with a single or very few hybridization and polyploidization events (de Blas et al., 2025). Yet, from this narrow genetic origin emerged two subspecies, six botanical varieties, and thousands of landraces differing in growth habit, seed size, pod morphology, and other traits. This paradox—an extreme genetic bottleneck followed by dramatic phenotypic expansion—raises a fundamental question: how is such rapid diversification possible from such limited genetic input?

One possible answer is polyploidy, which is often thought of as a driver of evolutionary innovation and crop domestication. Polyploidization, the acquisition of additional chromosome sets, can occur through genome duplication within a species (autopolyploidy) or through hybridization between different species (allopolyploidy). Polyploidy may occur via somatic doubling of chromosomes (of a hybrid, in the case of allopolyploidy), or the fusion of unreduced gametes (Gaeta & Pires, 2010).

The merger of two distinct diploid genomes can trigger various forms of genomic and regulatory instability, a phenomenon Barbara McClintock (1984) famously termed “genomic shock” (Renny‐Byfield & Wendel, 2014). In this study, the instability we observed can be attributed to the complexity of meiosis in a nucleus containing two complete chromosome sets from two different ancestral species.

In newly formed allotetraploids, different patterns of chromosome pairing can emerge. In some, chromosomes pair strictly with their homologs—that is, chromosomes derived from the same ancestral genome—resulting in diploid‐like genetic behavior. In other cases, known as segmental allotetraploids, some pairing also occurs between homoeologous chromosomes—that is the corresponding chromosomes from different ancestral genomes. This leads to the formation of multivalents, recombination between ancestral genomes, and genetic segregation that partially resembles that of autotetraploids (Gaeta & Pires, 2010; Gillies, 1989; Stebbins, 1971). Such multivalent pairing can promote genetic novelty by shuffling genome structure and altering allele dosage, thereby contributing to phenotypic diversity.

Peanut belongs to this latter category; its chromosomes mostly pair with their homologs, but occasionally with their homoeologs, classifying it as a segmental allotetraploid (Bertioli et al., 2019). The hybridization and genome doubling of *A. duranensis* (AA genome) and *A. ipaënsis* (BB genome) produced a tetraploid species with an AABB genome (2*n* = 4*x* = 40) (Bertioli et al., 2016, 2019, 2020; Fávero et al., 2006). Peanut retains almost complete copies of its ancestral genomes. Genomic *in situ* hybridization demonstrates clear differentiation between A and B chromosomes without detectable rearrangements or mosaics using that technique (Seijo et al., 2007). Genome size closely matches the sum of the diploid progenitors (*A. duranensis* ∼1.25 Gb, *A. ipaënsis* ∼1.56 Gb) (Bertioli et al., 2016; Samoluk et al., 2015), indicating no major expansion or contraction since polyploidization. Cultivated peanut exhibits mostly bivalent pairing and disomic inheritance, although tetravalent pairing and tetrasomic inheritance do occur. These irregularities have led to an autotetraploid structure in a small but significant proportion of the genome (Bertioli et al., 2016, 2019).

It is estimated that a large proportion of all angiosperms have undergone polyploidization, a process that can confer advantages such as heterosis, gene redundancy, breakdown of self‐incompatibility, and capacity for asexual reproduction (Comai, 2005; Soltis et al., 2015; Soltis & Soltis, 2012). These potential benefits have led to increasing interest in the role of polyploidy in facilitating plant domestication (Akagi et al., 2022; Salman‐Minkov et al., 2016). Several wild diploids (e.g., *A. stenosperma*, *A. villosulicarpa*) are known to have been cultivated in historic and prehistoric times, but only *A. hypogaea* fully developed the domestication syndrome and became a major global crop (Bertioli et al., 2011; supplementary note 1 in Bertioli et al., 2019; Krapovickas et al., 2007; Stalker & Wilson, 2016). This strongly suggests that polyploidization played a key role in peanut domestication.

To investigate whether polyploidy facilitated peanut domestication, we used a synthetic neoallotetraploid (IpaDur1) created by colchicine doubling of a hybrid between *A. duranensis* V 14167 and *A. ipaënsis* K 30076 (Fávero et al., 2006; Leal‐Bertioli et al., 2021). IpaDur1 closely recreates the genomic structure of the ancestral tetraploid from which peanut was domesticated. It has chromosomes resembling those of *A. hypogaea* (do Nascimento et al., 2018), but as an earlier generation polyploid, it shows greater genomic and phenotypic instability (Leal‐Bertioli et al., 2015, 2021). It is an excellent proxy for the prototype peanut of prehistory.

We advanced IpaDur1 lineages over 6 years in greenhouse conditions, selecting for divergent seed weight under pollinator‐free conditions. Diploid progenitors were included as controls. This simulated early stages of domestication while safeguarding against the experimental mischief of cross‐pollination by bees that would confound the experiment. A stable, strictly allotetraploid genome with purely disomic inheritance should show the same phenotypic uniformity as its diploid ancestors. Instead, we observed a “phenotypic boom” among IpaDur1 lineages, including traits that echo cultivated peanut diversity. Furthermore, the neoallotetraploid responded more strongly to artificial selection than its diploid parents. These findings provide experimental support for the hypothesis that polyploidy conferred a domestication advantage and helped drive the remarkable phenotypic diversification of *A. hypogaea*.

## RESULTS

### Enhanced response to artificial selection in IpaDur1 compared with diploid parents

**Figure 1:**
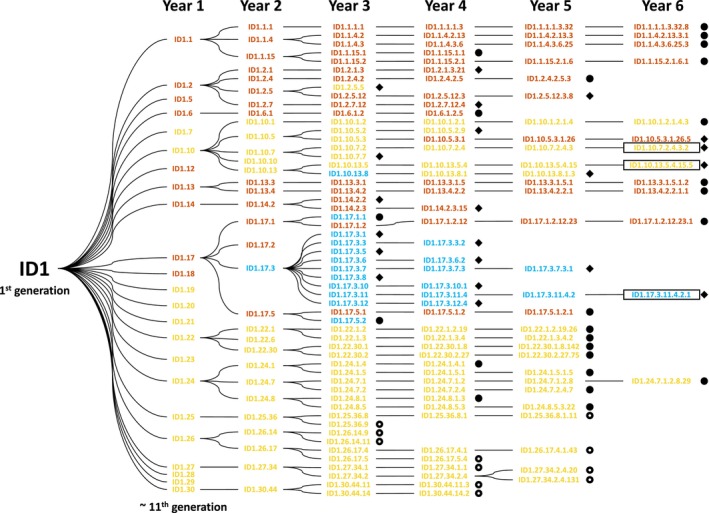
Lineage tree of IpaDur1 plants showing seed‐producing plants and the three sequenced genotypes (black rectangles). Circles, open circles, and rhombuses represent heavy, light, and average seed weight selection, respectively. Premature termination of lineages was caused by failure to produce viable seeds. Flower colors are coded: yellow (yellow), orange (orange), and orange/white/variegated/multicolored (light blue). Over 6 years, these lineages revealed striking spontaneous phenotypic variation, especially in flower color.

**Figure 2:**
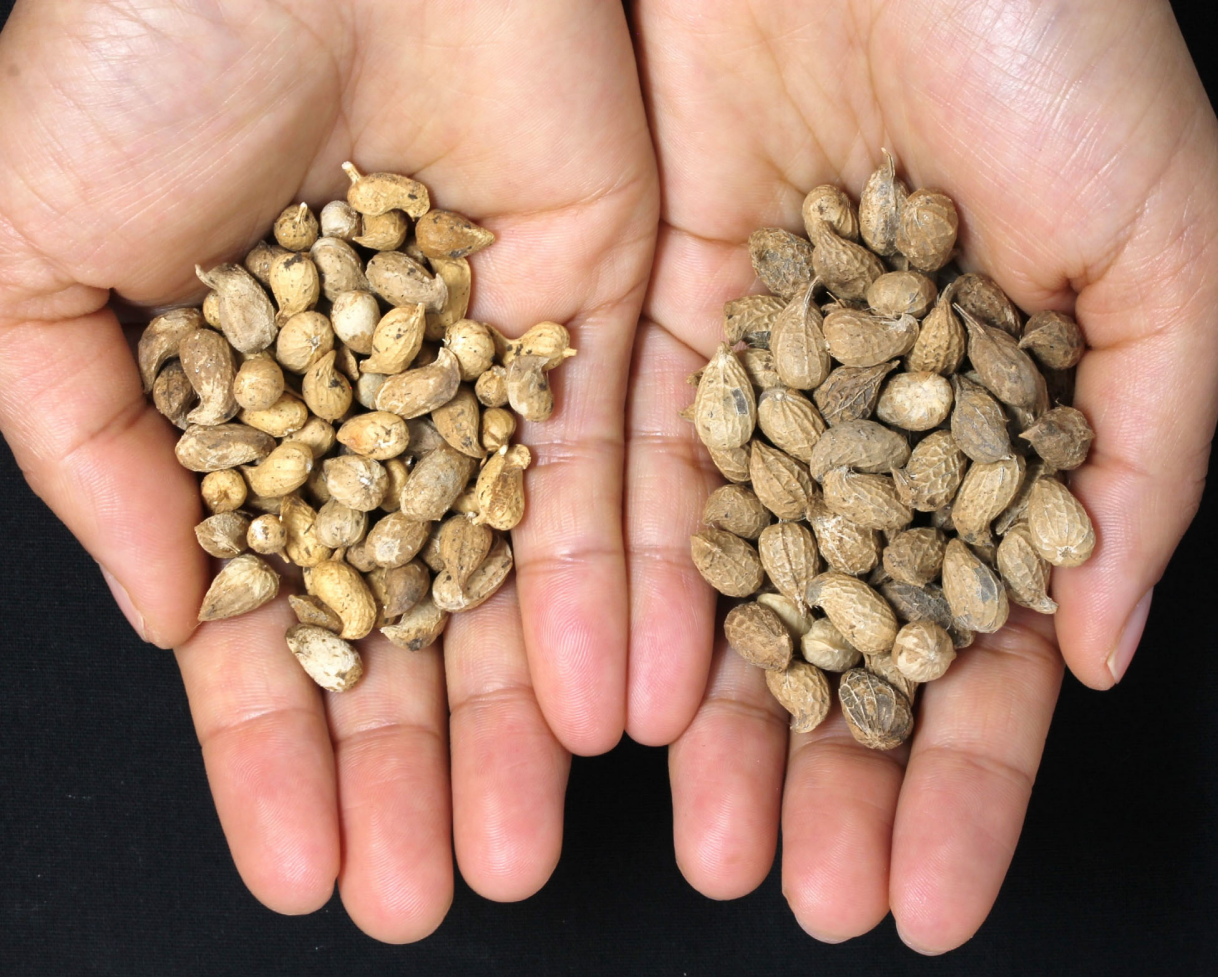
Neoallotetraploid IpaDur1 light and heavy seed weight selections. Pods are shown from two IpaDur1 genotypes: a light seed weight selection (ID1.25.36.8.1, left) and a heavy selection (ID1.22.30.1.8, right). The light selection produced smaller, smooth pods resembling *Arachis duranensis*, while the heavy selection produced larger, reticulated, rounder pods resembling *Arachis ipaënsis*.

To test whether polyploidy conferred an advantage for domestication, we subjected IpaDur1 lineages—starting at roughly the 13th generation of selfing—and their diploid progenitors to 3 years of artificial selection for divergent seed weights (Figure 1; Figures S1 and S2). From years 3–5 of our experiment, all three populations underwent targeted selection based on contrasting seed weight. Nearly always, only one seed per plant was advanced to the next generation, creating strong selection pressure. IpaDur1 lineages exhibited a much stronger response than either of its diploid parents (Figure 2; Figure S3; Table S1). Light seed selection resulted in lower seed weight, while heavy seed selection produced larger seeds than average weight controls—a pattern not seen in *A. duranensis* and only seen to a very small degree in *A. ipaënsis*.

**Figure 3:**
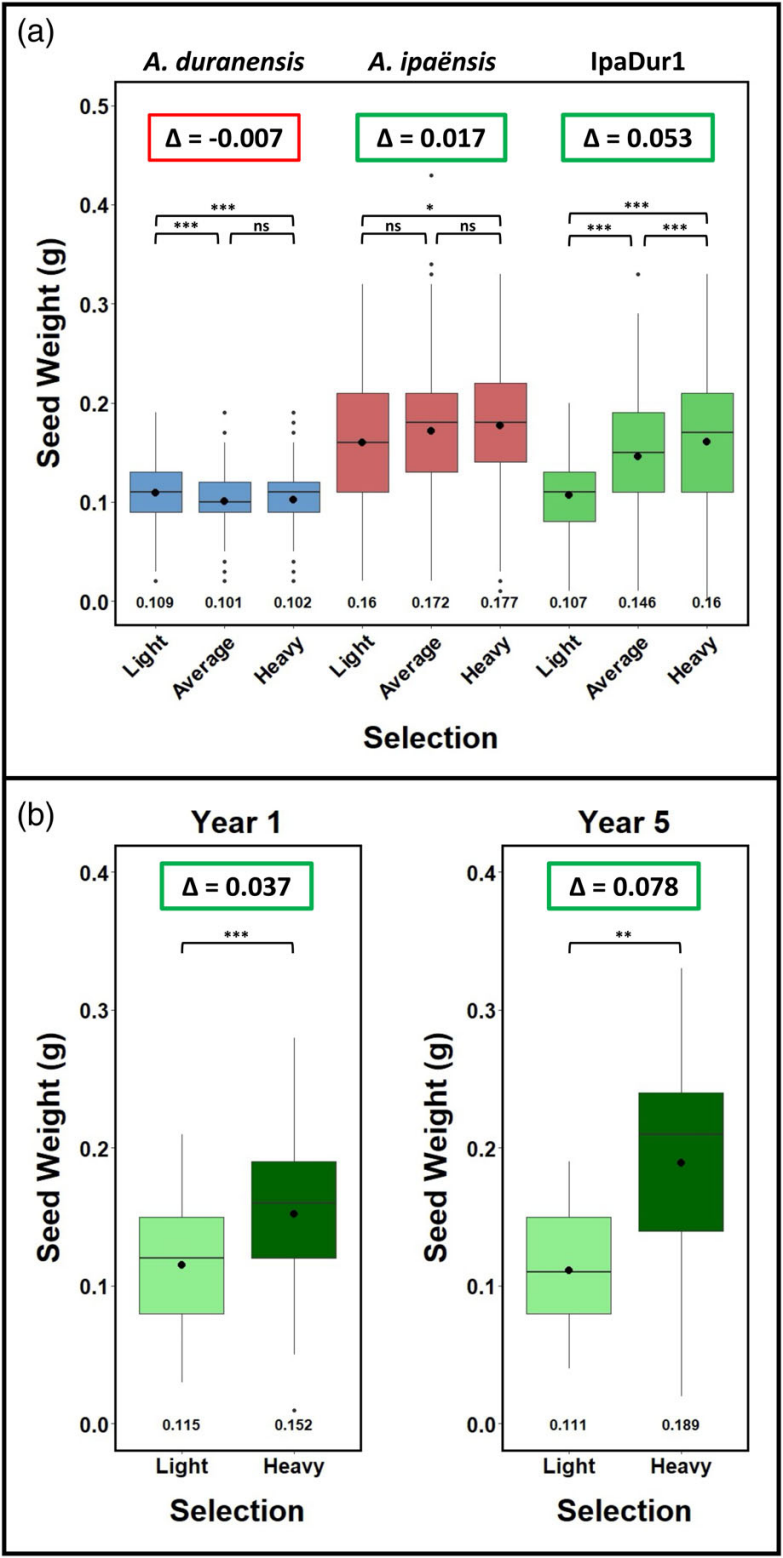
IpaDur1 shows greater response to selection than its wild diploid parents. Box plots show seed weight variation across years of selection for *Arachis duranensis* (blue), *Arachis ipaënsis* (red), and IpaDur1 lineages (green), under light, average, and heavy seed weight selections. Heavy black dots and numbers represent average seed weight. Δ indicates the difference between heavy and light selection averages, with the red box indicating ineffective and green box indicating effective selections. Significance was tested using Kruskal–Wallis with Dunn's post hoc test (*P < 0.05, **P < 0.01, ***P < 0.001). “ns” denotes non‐significant differences. (a) IpaDur1 seed weights of light and heavy selections resemble those of *A. duranensis* and *A. ipaënsis*. IpaDur1 lineages showed greater responsiveness to artificial selection than the wild diploid parents. (b) The effectiveness of selection in IpaDur1 comparing seed weights of light and heavy selections in years 1 and 5.

To account for yearly environmental effects, we combined data across years and calculated the seed weight difference between heavy and light selections (Figure 3a). IpaDur1 showed an average seed weight difference of 0.053 g/seed (0.160 g heavy versus 0.107 g light), while *A. ipaënsis* showed only 0.017 g/seed and *A. duranensis* showed a negative difference (−0.007 g/seed). Notably, heavy selections in IpaDur1 were comparable to *A. ipaënsis*, while light selections produced seeds lighter than the light selection of *A. duranensis*, demonstrating transgressive segregation.

Similar patterns were seen for maximum seed weights (Figure S4; Table S1). The effect of artificial selection in IpaDur1 was surprisingly strong after just 3 years (Figure 3b). Pod traits also responded: heavier seeds were associated with denser, rounder pods (Figure S5; Table S1).

Multiple linear regression confirmed a strong relationship between selection regime and outcomes including seed weight, maximum seed weight, pod area, pod perimeter and pod circularity. IpaDur1 outperformed diploid parents in adjusted R‐squared values, indicating stronger and more reliable trait prediction (Table S2). All traits satisfied normality assumptions. Together, these results show that IpaDur1 responded more robustly to artificial selection than its diploid progenitors.

### Determining genome compositions using genotyping and whole‐genome sequencing

Our genotyping analysis with “fitPoly” (Voorrips, 2025; Voorrips et al., 2011) allowed us to determine all possible dosages of *A. duranensis* and *A. ipaënsis* alleles in the tetraploid state: BBBB, ABBB, AABB, AAAB, and AAAA (Table S3). This allowed the routine identification of unbalanced genomic compositions, which is not possible in studies that use methods and software restricted to three genotypic groupings such as the basic form of Axiom Analysis Suite (Thermo Fisher Scientific, Waltham, MA, USA). The “fitPoly” package in RStudio provided allele scoring on a true tetraploid scale, allowing detection of five distinct dosage states. In contrast, software limited to three genotype calls often artifactually misclassifies ABBB and AAAB regions, causing erratic switching between genotype calls and reducing the reliability of genome composition analysis.

However, one limitation of the “fitPoly”‐based approach was its inability to detect compositions that fall outside of standard tetraploid dosage classes. For example, in line ID1.10.7.2.4.3.2, “fitPoly” misclassified an aneuploid AAAAB composition on chromosome set 06 as a standard AAAB. This likely contributed to reduced marker retention in chromosome set 06 after filtering, as the additional chromosome led to inconsistent scoring and marker exclusion (Table S3). Nevertheless, sufficient markers were retained to visualize genome compositions accurately, and for consistency of methods across the analysis, we retained the same genotyping pipeline throughout.

**Figure 4:**
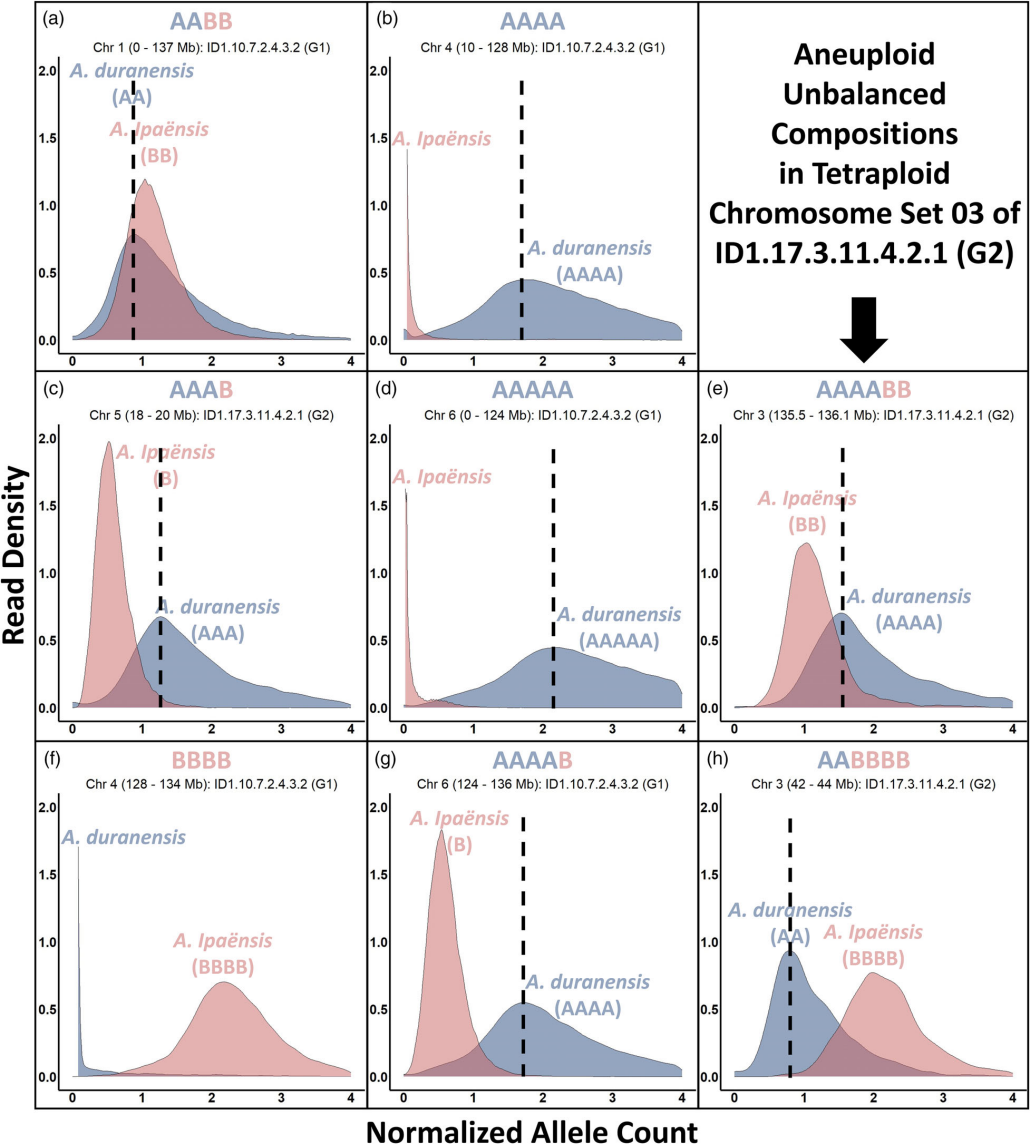
Analysis of genomic compositions using whole‐genome sequencing. Genomic profiles of two selected IpaDur1 genotypes—ID1.10.7.2.4.3.2 (G1 group) and ID1.17.3.11.4.2.1 (G2 group)—are shown as frequency distributions of normalized A and B allele counts across selected chromosomal segments. Panels illustrate a range of genomic compositions: balanced AABB (a), unbalanced AAAA (b), unbalanced AAAB (c), unbalanced BBBB (f), and aneuploid unbalanced AAAAA (d), AAAABB (e), AAAAB (g), and AABBBB (h). This approach infers allele dosage and genome composition directly from sequencing read frequencies and is not constrained by the assumptions of SNP‐based genotype calling algorithms, making it well suited to the analysis of complex neoallopolyploid genomes. Normalized A allele counts are consistently slightly underestimated relative to their true values (dashed lines) and display lower density, likely due to the normalization procedure.

The use of Illumina sequencing and subgenome‐specific allele quantification overcame these limitations. By mapping to a reference genome and analyzing subgenome‐specific allele counts, we were able to independently determine genome composition across chromosome sets without relying on fixed genotype classes. This sequencing‐based approach confirmed the genotyping results in most cases but also revealed additional, unexpected dosage states—including aneuploid unbalanced AAAAA, AAAAB, AAAABB, and AABBBB configurations (Figure 4)—that were missed or misclassified by SNP‐based methods. These results demonstrate the power of whole‐genome sequencing to resolve complex genomic structures.

### Dynamic and unbalanced genome compositions in IpaDur1

**Figure 5:**
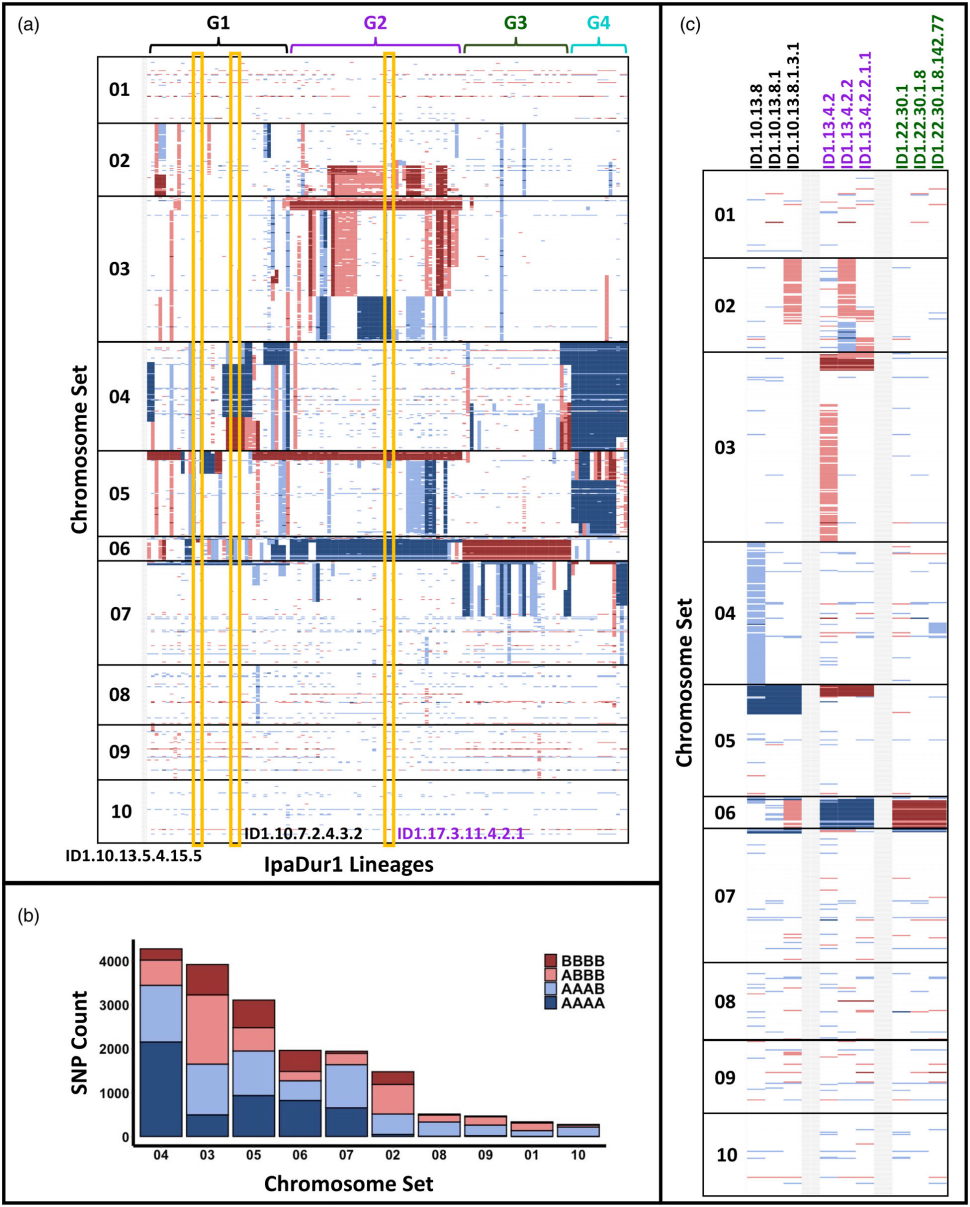
Graphical representation of IpaDur1 lineages' genomic compositions by genotyping analysis. Balanced segments (AABB) are shown in white. Unbalanced compositions are color‐coded: BBBB in dark red, ABBB in light red, AAAB in light blue, and AAAA in dark blue. (a) Color‐coded matrix showing SNP‐based genotypes of IpaDur1 plants grouped in lineages (columns), with markers ordered by physical position on the *Arachis ipaënsis* genome (gnm1) along the chromosome sets (rows). Sequenced plants are highlighted with yellow boxes. (b) Bar plot showing normalized SNP counts for unbalanced genomic compositions across chromosome sets. Shifts toward *A. duranensis* are prominent in sets 04 and 07, while greater shifts toward *A. ipaënsis* are observed in sets 02 and 03. (c) Genome composition shifts over generations in three lineages representing three groups: G1 in black, G2 in purple, and G3 in green. BBBB and AAAA states remain stable, while ABBB and AAAB states are unstable across generations. IpaDur1 lineages display a high frequency of unbalanced genomic segments, with active homoeologous exchange shaping genome composition across generations.

We used a color‐coded matrix to visualize chromosomal compositions in homeologous “sets” (Figure 5a; Table S3). Set 01 refers to B01 and A01; set 02 refers to B02 and A02, following the simple numerical correspondence except for chromosomes 07 and 08: set 07 refers to B07 and parts of A07 and A08; chromosome set 08 refers to B08 and different parts of A07 and A08. This deviation results from reciprocal translocation and rearrangements in the A genome evolutionary lineage, as described by Bertioli et al. (2016).

Significant deviations from balanced AABB compositions were observed, including BBBB, ABBB, AAAB, and AAAA patterns—especially in sets 04, 03, 05, 06, 07, and 02 (Figure 5b). Chromosome sets 04 and 07 showed shifts toward *A. duranensis*, while sets 02 and 03 shifted toward *A. ipaënsis* (Figure 5b).

**Figure 6:**
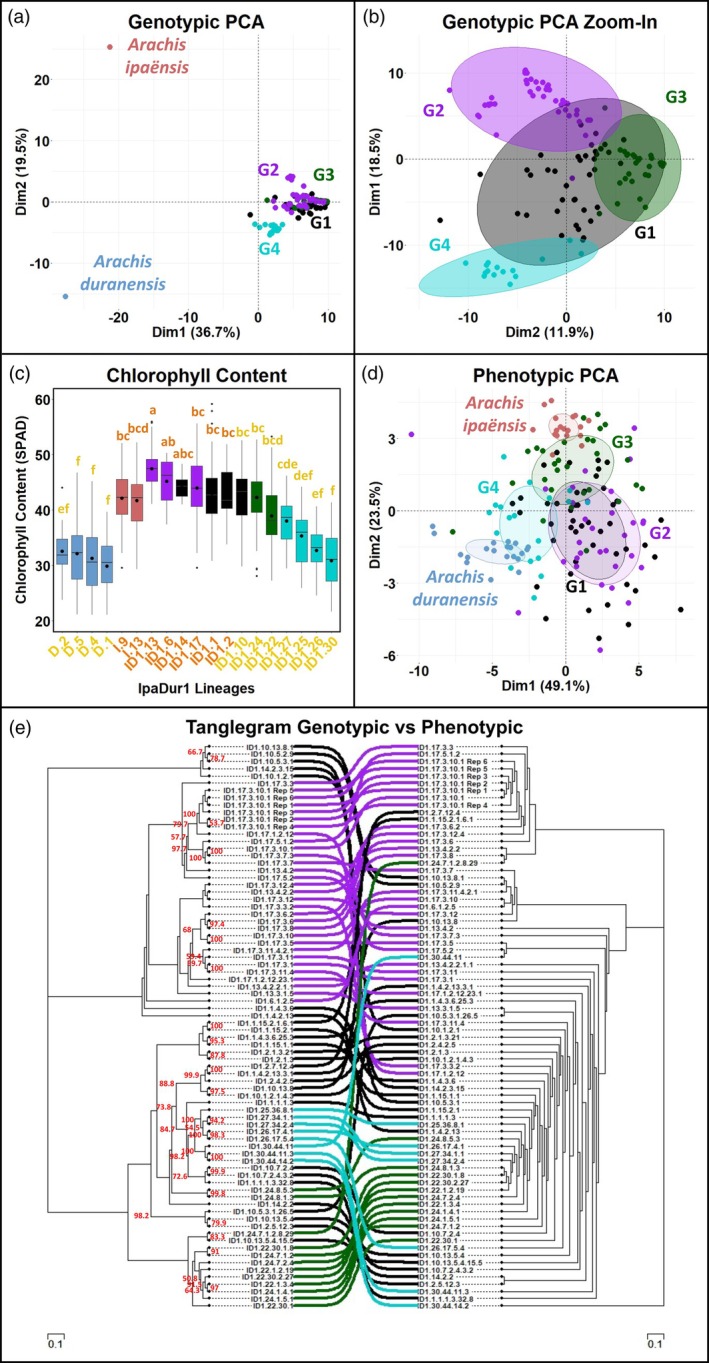
Correlation of the genetic and phenotypic diversity generated in IpaDur1 lineages. (a) Principal component analysis (PCA) of genotypic data shows clear separation between wild diploids (*Arachis duranensis*, blue; *Arachis ipaënsis*, red) and IpaDur1 lineages. IpaDur1 genetic groups (G1–G4) are color‐coded and separated by unbalanced genomic compositions in specific chromosomes. (b) A “zoom in” PCA of IpaDur1 lineages only (diploids removed) highlights clearer separation among genetic groups G1–G4. (c) Leaf chlorophyll content (SPAD) in *A. duranensis* (blue boxes), *A. ipaënsis* (red boxes), and IpaDur1 lineages, boxes color‐coded by genetic group. Letters above boxes indicate Tukey groupings (*P* < 0.05); orange and yellow letters mark IpaDur1 genotypes with predominantly orange or yellow flowers, respectively. Black dots show group means. Data were collected in generations 4 and 6, organized by first‐generation progenitors. Levene's test (*P* = 0.28), ANOVA (*P* < 0.001), and Shapiro–Wilk test (*P* = 0.889) confirmed the assumptions of variance and normality. (d) PCA of phenotypic data shows clear diploid separation along PC2, with IpaDur1 lineages displaying a broad phenotypic spread. Group G3 trends toward *A. ipaënsis* phenotypes, while G4 trends toward *A. duranensis*, reflecting underlying genomic shifts. (e) Tanglegram comparing a genetic distance tree (left, based on SNP data) with a phenetic tree (right, based on seed and pod traits) for IpaDur1 lineages. Group‐level correspondence and crossover highlight the correlated relationship between genotype and phenotype.

Based on lineage information and genomic compositions, we classified IpaDur1 plants into four genetic groups (G1–G4) (Figures 5a and 6a; Figure S6). G1 includes progenies of ID1.1, ID1.2, ID1.10, and ID1.14 and had diverse genome shifts toward both progenitors; G2 comprises progenies of ID1.6, ID1.13, and ID1.17 and had major shifts toward *A. ipaënsis* on sets 03 and 05 and toward *A. duranensis* on set 06; G3 consists of progenies of ID1.22 and ID1.24 and showed shifts predominantly toward *A. ipaënsis*, and G4 consists of progenies of ID1.25, ID1.26, ID1.27, and ID1.30 and predominantly shifts toward *A. duranensis*.

We tracked genomic compositions across generations to assess stability. In lineages genotyped over three or more consecutive generations, only BBBB and AAAA states remained stable. In contrast, ABBB and AAAB states were not stable over generations, sometimes reverting to balanced AABB or shifting further to BBBB and AAAA states (Figure 5c). This instability persisted even after 17 generations from the polyploidy event, consistent with ongoing genomic flux.

Principal component analysis (PCA) of genotyping results placed IpaDur1 lineages between the diploid progenitors, with PC1 (37% variance) distinguishing ploidy and PC2 (20%) distinguishing genomic composition (Figure 6a). G4 grouped toward *A. duranensis* and G2/G3 toward *A. ipaënsis*, reflecting the overall direction of genome shifts (Figure 6b).

### Sequencing identifies complex genome compositions and ploidy variants

**Figure 7:**
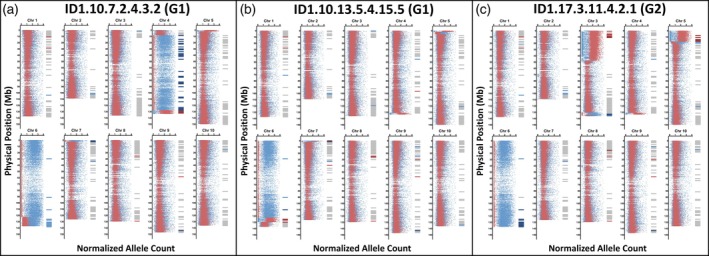
Sequencing validation of genotyping results in selected IpaDur1 plants. (a–c) Results for plants ID1.10.7.2.4.3.2 (a), ID1.10.13.5.4.15.5 (b) from group G1, and ID1.17.3.11.4.2.1 (c) from group G2. In each chromosome sub‐panel, the left half shows allele density distributions from whole‐genome sequencing; the right half shows genotyping results. *Arachis duranensis* alleles are plotted in blue, *Arachis ipaënsis* in red in the sequencing. In SNP‐based genotyping, genomic dosage imbalances are color‐coded: BBBB regions in dark red, ABBB in light red, AAAB in light blue, and AAAA in dark blue. Balanced genomic compositions (AABB) are shown in gray. Clear distortions are visible in panel a (chromosome sets 04 and 06), panel b (set 06), and panel c (sets 03, 05, and 06). SNP‐based genotyping and sequencing results are concordant.

We performed Illumina sequencing and subgenome‐specific allele quantification on three IpaDur1 plants, representing different genotypic groups, to validate genotyping results and explore chromosomal composition in greater detail (Figures 4a–h and 7a–c). Sequencing largely confirmed genotyping results but uncovered additional cases of aneuploidy AAAAA, AAAAB, AAAABB, and AABBBB anomalies that had gone misidentified or undetected by genotyping.

In chromosome set 03, one G2 plant (ID1.17.3.11.4.2.1) exhibited a striking transition: a 40 Mb BBBB region followed by an AABBBB segment, then by a BBBB followed by a balanced AABB region spanning 80 Mb, and finally by a terminal AAAA region followed by an AAAABB segment. In set 06, another plant showed an AAAAA composition with a terminal AAAAB stretch, which had been misclassified as AAAB in SNP‐based genotyping. This highlights a limitation of SNP‐based genotyping methods that assume fixed ploidy, and underscores the value of whole‐genome sequencing for accurately resolving unexpected chromosomal configurations.

**Figure 8:**
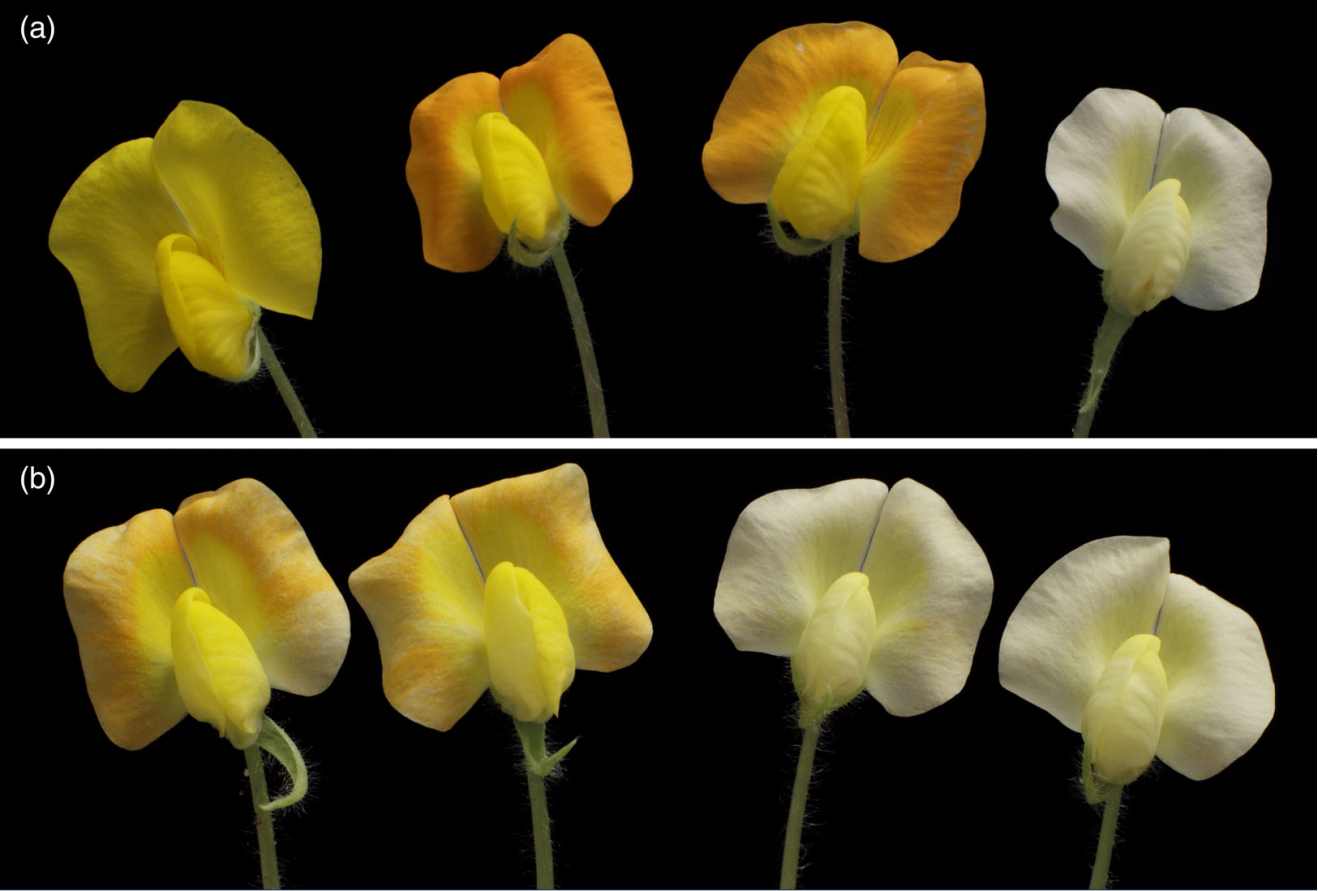
Emergence of new flower color phenotypes in IpaDur1 lineages. New flower color phenotypes spontaneously emerged in IpaDur1 lineages, distinct from the diploid parents *Arachis duranensis* V 14167 (yellow) and *Arachis ipaënsis* K 30076 (orange). (a) Diverse flower colors from four different plants, including yellow (original hybrid color), two shades of orange, and white, from left to right. (b) Displays examples of variegated and white flowers, all from the same plant.

*Arachis duranensis* V 14167 has yellow flowers, *A. ipaënsis* K 30076 has orange. In the IpaDur1 plants, the flower phenotype correlated with genome composition at the top of chromosome set 05, which harbors flower color genes (Bertioli et al., 2019). AABB plants produced yellow flowers, consistent with the known dominance of the yellow allele over orange. BBBB plants exhibited either orange flowers—reflecting their *A. ipaënsis* genomic composition—or white flowers, likely caused by gene silencing due to superdosage imbalance (Figure 8). One plant within the white‐flowered lineage also showed an exceptional case of flower shape variation (Figure S7a). AAAA plants almost always had yellow flowers reflecting their *A. duranensis* genome composition, although in one lineage, a transient white‐flowered phenotype was observed (Figures 1 and 7a–c; Table S3).

These findings highlight the scale and persistence of genomic instability in IpaDur1—even after 17 generations of selfing.

### Phenotypic diversity expands in IpaDur1 and correlates with parental genome balance

Individual plants of IpaDur1, *A. duranensis*, and *A. ipaënsis* were phenotyped for multiple traits over 6 years, including floral characteristics, seed and pod yield components, chlorophyll content, and detailed pod morphology (see “Methods” section for full list). Initial generations of IpaDur1 had yellow flowers, but later generations exhibited a spectrum of flower colors including orange, white, and variegated (Figures 1 and 8). Some plants showed intraindividual flower variation and rare changes in flower shape (Figure S7a–d).

Chlorophyll content, measured by SPAD, was stable in diploid parents but highly variable in IpaDur1. Higher chlorophyll content was associated with orange flowers (resembling *A. ipaënsis*) and lower values with yellow (like *A. duranensis*) (Figure 6c).

PCA of phenotypes (Figure 6d) showed that IpaDur1 plants scattered across the phenotypic space defined by their diploid parents, with distinct contributions from pod size, shape, and number of seeds (Figures S8 and S9). Seed weight, the target of selection, mainly contributed to a separate axis (dimension 4). Phenotypic variations corresponded with genomic changes caused by homeologous recombination. We also observed differences in growth habit. Generally, lineages with a genomic shift toward *A. duranensis* displayed phenotypes more similar to *A. duranensis*, while those with a shift toward *A. ipaënsis* exhibited traits resembling *A. ipaënsis*. Group G4, with a shift toward *A. duranensis*, showed light green leaves, yellow flowers, smoother pods and smaller seeds (Figures 2 and 6c; Figure S10). Groups G2 and G3, characterized by shifts toward *A. ipaënsis*, exhibited dark green leaves, orange or yellow flowers, reticulated pods, and for G3, large seeds. The difference in seed sizes between G2 and G3 may be explained by a genetic factor that confers large seeds that resides at the end of chromosome B06 (Foncéka et al., 2012; Tossim et al., 2020). While G3 has shifted toward *A. ipaënsis* in this region, G2 has shifted to *A. duranensis* (Figure 5a).

This congruence between genotype and phenotype was confirmed both by PCA and clustering analyses (Figure 6a–e). The tanglegram shows that most genotypes occupy similar positions in the genotypic and phenotypic trees, with only minor discrepancies. The parallel structure of genomic and phenotypic diversity strongly supports an underlying connection. Together, these data show that polyploid instability and homoeologous exchange produced diverse and heritable variation, enabling strong responses to selection from a genetically uniform starting point.

## DISCUSSION

We show that neoallotetraploid peanuts derived from a single hybridization and polyploidization event generate heritable phenotypic diversity and respond strongly to artificial selection (Figure 3). Remarkably, this variation arises quickly and spans traits central to peanut domestication, including seed size, pod morphology, and chlorophyll content. The central paradox—that domestication and diversification occurred in the absence of initial genetic diversity—effectively disappears.

The variation we observe is not random. With our controlled advancing of multiple IpaDur1 lineages, we were able to observe the generation of phenotypic diversity in greater range and detail than before (Figures 1, 2, 6c–e, and 8). Traits such as seed size, pod shape, leaf tone, and flower color consistently correspond with genomic shifts toward one or the other diploid parent. Overall, lineages whose genome shifted toward *A. ipaënsis* tend to have larger seeds, more reticulated pods, darker leaves, and orange flowers; those that shifted toward *A. duranensis* show smaller seeds, smoother pods, lighter leaves, and yellow flowers. These patterns recur across multiple traits and lineages.

Inferring causation is fraught with difficulty—as the Scottish Enlightenment philosopher Hume (1748) warned—so science pragmatically relies on experimentation, empirical criteria, and probabilistic reasoning to guide such judgments. One influential set of criteria, proposed by epidemiologist Hill (1965), outlines conditions under which causation may reasonably be inferred from observational data. Considering these criteria, the most compelling observation in our study is this consistent directional parallelism between genotype and phenotype: as genomic balance shifts toward one parent, the traits follow. This congruence strongly supports causality.

Flower color provides the clearest and most specific example. The relevant genetic locus is known to reside on chromosome set 05, where the *A. duranensis* yellow allele is dominant over *A. ipaënsis*' orange allele (Bertioli et al., 2019). Plants with AABB or AAAA dosage in this region had yellow flowers, consistent with the dominance of yellow. BBBB plants showed orange flowers—reflecting their *A. ipaënsis* ancestry—or white or variegated flowers, likely due to gene silencing caused by dosage imbalance. These patterns align with genetic expectations and satisfy several of Bradford Hill's criteria: plausibility, gradient, specificity, and consistency.

It is widely believed that polyploidy is favored in plant domestication, although evidence is not as clear‐cut as often assumed (Hilu, 1993; Meyer et al., 2012). Proposed advantages of polyploidy include gene redundancy, buffering of deleterious alleles, and fixed hybrid vigor (Comai, 2005; Selmecki et al., 2015; Soltis & Soltis, 2012). However, polyploidy also brings genetic penalties: reduced fertility, genomic instability, and difficulty fixing favorable alleles (Soltis et al., 2014). A belief in the advantages of polyploidy in domestication should be tempered by the modest difference in polyploid frequency between crops and wild plants (30% versus 24%) (Salman‐Minkov et al., 2016; Wood et al., 2009). The development of diploid potatoes with yields comparable to traditional tetraploid cultivars further cautions against the assumption that polyploidy is intrinsically superior (Jansky et al., 2016; MPIC Potato Research Report, 2023). As far as we are aware, prior empirical demonstrations of polyploidy's value for domestication have been lacking. Wang et al. (2024) come close, offering a compelling case that homoeologous exchange contributed to the domestication of *Brassica* polyploids, based on associations between exchange regions and agronomic traits. However, strictly speaking, their study is retrospective and correlational; it does not directly test the capacity of polyploidy to generate selectable phenotypic diversity that contributes to domestication.

We believe the case for a polyploid advantage in peanut domestication is exceptionally strong. Although several diploid *Arachis* species were cultivated earlier in prehistory, only the polyploid form became fully domesticated and a major global crop (see supplementary note 1 of Bertioli et al. (2019)). The diploids lapsed from cultivation, remaining at most as proto‐domesticates (e.g., the Atlantic coast form of *A. stenosperma*) or, in the case of *A. villosulicarpa*, expressing the domestication syndrome much more weakly. Together, these observations strongly suggest that polyploidy conferred significant advantages. But what were these advantages? Polyploid peanuts have been shown to differ from diploids due to nucleotypic effects, such as increased cell and plant size, higher levels of photosynthetic pigments, and altered transpiration profiles. Surprisingly, however, the seed size of initial generation neoallotetraploid peanuts remains similar to their diploid parents (Leal‐Bertioli et al., 2012, 2017). This study aimed to test the response of IpaDur1 lineages to artificial selection compared with the diploid parents. We focused on seed size because cultivated peanut has larger seeds than the wild species and their derived neoallotetraploids. The markedly stronger response to selection that we observed in the IpaDur1s would clearly have favored domestication in the proto‐peanut of prehistory. Even more strikingly, several of the spontaneously emerging phenotypes—variation in flower color, pod morphology, and leaf pigmentation—mirror traits that distinguish subspecies and landraces of *A. hypogaea* today. It appears that even from a genetically uniform starting point, key aspects of peanut domestication are naturally re‐emerging.

However, before drawing too strong parallels to modern peanut, it is important to recognize that the genomic profiles of the IpaDur1s differ markedly from those of the crop today. While IpaDur1 lines frequently carry large‐scale BBBB and AAAA chromosomal regions, these are smaller in *A. hypogaea* (Bertioli et al., 2019). In the Tifrunner reference genome, only 14.8 Mb of the A genome and 3.1 Mb of the B genome are AAAA or BBBB. These regions are mainly at the distal ends of chromosomes, including the lower regions of chromosome sets 02, 04, 06, and the upper regions of chromosome set 05 (Bertioli et al., 2019). This is minimal compared with, for example, ID1.17.3.11.4.2.1, which exhibits over 40 Mb of BBBB genome composition solely in chromosome set 03. These differences suggest that during peanut evolution, large‐scale unbalanced BBBB and AAAA carried fitness costs and were selected against, likely due to disruption of the “fixed hybrid” state that underpins polyploid peanut vigor. Such imbalances may reduce fertility or compromise growth. This scenario is consistent with the “polyploid ratchet” model (Gaeta & Pires, 2010), which proposes that in early polyploid species, large‐scale genomic instability, allele loss, and dysfunction can accumulate, ultimately compromising lineage survival. This ratchet constrains the viable genomic configurations available to polyploids in the long term.

Our observations in flower color offer one example of how these constraints may have operated: nearly all white‐flowered IpaDur1 plants had BBBB structure at the flower color locus, whereas only one AAAA plant displayed a transient white‐flower phenotype. This asymmetry is consistent with previous findings that the B subgenome not only exhibits higher gene expression but also has more transposable elements than the A subgenome (Bertioli et al., 2016, 2019). A shift to BBBB dosage may therefore increase the risk of regulatory disruption—through both superdosage and transposon‐mediated silencing—leading to gene silencing. The occurrence of deformed white flowers in a BBBB chromosome set 05 IpaDur1 plant adds weight to the link between gene dosage imbalance and developmental dysfunction. Peanut domestication likely favored lineages with more stable and viable genomic configurations—those that maintained gene expression and preserved the functional balance between subgenomes. This, in turn, could explain why in cultivated peanut deviations from the balanced AABB composition at the distal ends of chromosome arms are smaller and overwhelmingly AAAA (Bertioli et al., 2019). These regions may have fixed to the A genome not because of enhanced function, but because they better preserved gene expression and avoided silencing.

Once BBBB and AAAA genome compositions occur, they are fixed within a self‐pollinating lineage. In contrast, ABBB and AAAB compositions are more dynamic, with the potential to revert to the balanced AABB state or fix into BBBB and AAAA configurations (Figure 5a,c). This implies that although BBBB and AAAA compositions were likely selected against during peanut evolution, ABBB and AAAB genome compositions may be occasional transient states in the modern peanut genome, with ongoing fluctuations between balanced AABB and unbalanced ABBB or AAAB states still occurring.

Our findings show that even a single polyploidization event—an extreme genetic bottleneck—can generate a boom of heritable, selectable diversity. Homoeologous recombination and dosage‐dependent gene expression and silencing appear to be key drivers. At the same time, large‐scale genomic imbalances common in early generation polyploids (such as AAAA and BBBB) are largely absent in the modern crop. This pattern suggests strong historical selection for genomic stability. This raises the next question—to what extent does this instability still persist? In the third and final study of this series, we address this question directly, examining to what degree spontaneous chromosomal changes continue to occur in modern cultivated peanut of pure pedigree (Lamon et al., 2025).

## METHODS

### Plant material and selection criteria

A neoallotetraploid was generated by hybridizing *A. ipaënsis* K 30076 (BB genome, female parent) with *A. duranensis* V 14167 (AA genome, male parent), followed by inducing chromosome doubling through colchicine treatment (Fávero et al., 2006). The resulting neoallotetraploid is herein called IpaDur1. IpaDur1 was created in 2004/5, and underwent several generations without any trait selection. When this experiment started, plants were around the 11th generation of selfing, and we started counting years from the start of the experiment.

In this experiment we advanced lineages of IpaDur1 plants, alongside its wild diploid parents, for 6 years in the greenhouse. During the third to fifth years, IpaDur1, *A. duranensis* and *A. ipaënsis* lineages, underwent selection based on seed weight to evaluate their response to selection, based on three categories: heavy, average, and light seed weight.

To track each individual at each generation, we used a naming convention pedigree in single seed descent. Progenies of IpaDur1 (ID1), *A. duranensis* (D) and *A. ipaënsis* (I) have numbers that indicate the particular generation and pedigree. For example, in ID1.10.13.5.4.15.5, the prefix ID1 denotes IpaDur1, and the six separate numbers indicate it belongs to the sixth year of advancement. Each preceding year's seed number and parent plant are noted, like ID1.10 representing the plant #10 of IpaDur1 in the first year of the experiment.

By the third experimental year, differences in seed size among plants were already noticeable. Consequently, some IpaDur1 lineages with larger seed sizes (e.g. progenies of ID1.1, ID1.6, ID1.13, ID1.22, ID1.24) were consistently selected for heavy seed weight, while others with smaller seed sizes (progenies of ID1.25, ID1.26, ID1.27, ID1.30) were selected for light seed weight. Progenies of ID1.14 were consistently selected for average seed weight. Progeny of other IpaDur1 plants (ID1.2, ID1.10, ID1.17) had varying selections, with some lineages chosen for heavy seed selection, others for average, and others for light seed selection.

### Genotyping and sequencing

DNA was extracted from selected genotypes of *A. duranensis* (AA genome), *A. ipaënsis* (BB genome), and IpaDur1 lineages (AABB genome) using young leaves collected from plants that were two to 6 months old, following the DNeasy Plant Mini Kit protocol (Qiagen, Hilden, Germany). Additional control samples consisted of nine DNA mixtures representing AABB, AAAB, and ABBB genome compositions, with three mixtures each at ratios of 1:1, 3:1, and 1:3 of *A. duranensis* to *A. ipaënsis*, respectively. Genotyping was performed using the Axiom *Arachis* 48K SNP array v2 (Affymetrix, Santa Clara, CA, USA) (Clevenger et al., 2017; Korani et al., 2019; Pandey et al., 2017). Additionally, the genotypes ID1.10.7.2.4.3.2, ID1.10.13.5.4.15.5, and ID1.17.3.11.4.2.1 were sequenced at 30x coverage using Illumina Whole Genome Sequencing (WGS) NovaSeq (PE150) technology (Novogene Co, Sacramento, CA, USA).

### Genotypic data analysis

Raw. CEL files from the Axiom *Arachis* 48K SNP array v2 were processed with Axiom Analysis Suite 2022 to generate a summarized signal intensity file, which was subsequently imported into RStudio for further analysis (R Core Team, 2023). SNP markers for both diploid and tetraploid samples were initially scored on a tetraploid scale (0–4), representing allele dosages in segmental allotetraploid peanuts, using the “fitPoly” package. However, due to the inclusion of diploid controls, several molecular markers were scored on a diploid scale (Table S4). Therefore, scoring was repeated for IpaDur1 plants and DNA mixtures without diploid controls to ensure scoring on a tetraploid scale (Table S5).

A series of filtering steps was applied to the integrated dataset in Excel (Microsoft, Redmond, WA, USA). Initially, markers with scores across all individuals were retained. Markers were then selected based on their conformity to expected scoring in the control DNA mixes. Markers were preserved if the 1:1 DNA mixtures (AABB) had a modal score of 2, indicating subgenome homozygosity for opposite alleles. Subsequently, markers were retained if the 3:1 (AAAB) and 1:3 (ABBB) mixtures displayed modal scores of either 3 or 1, reflecting triplex compositions, depending on whether the A or B allele was counted. Additionally, markers were included if at least 25% of IpaDur1 plants showed a score of 2, indicating the expected balanced AABB genome composition. This filtration process prioritized markers aligned with the physical positions of the *A. ipaënsis* genome (version gnm1), reducing the dataset to 859 markers.

To standardize scores, only *A. duranensis* (A alleles) was counted, assigning a score of 3–3:1 mixtures (AAAB) and a score of 1–1:3 mixtures (ABBB). As expected, after these filtrations, *A. duranensis* plants exhibited consistently higher modal scores compared with *A. ipaënsis*. To convert to a tetraploid scale, diploid control scores were adjusted, with *A. duranensis* set to 4 and *A. ipaënsis* to 0 (Table S3). Using “ggplot2,” (Wickham et al., 2025) a color‐coded matrix was constructed based on the physical positions of markers along the *A. ipaënsis* chromosomes to visualize genome compositions in the different IpaDur1 plants. This visualization highlighted BBBB, ABBB, AAAB, and AAAA compositions within different chromosomes of different IpaDur1 plants.

To quantify BBBB, ABBB, AAAB, and AAAA, chromosome compositions across the genome, a summary bar plot was created using “ggplot2.” Genotyping calls were normalized by the number of SNP markers per chromosome across the IpaDur1 lineages. A PCA was conducted using the “adegenet” (Jombart & Ahmed, 2011), “ade4” (Dray et al., 2007) “FactoMineR” (Lê et al., 2008) and “factoextra” (Kassambara & Mundt, 2020) packages to assess genetic relationships. A secondary PCA, excluding diploid parents, further delineated separations among IpaDur1 lineages along the first two principal components, visualizing genetic variation across the samples. Finally, a neighbor‐joining phylogenetic tree with 1000 bootstrap replicates was constructed using “adegenet,” “ape” (Paradis & Schliep, 2019), and “poppr” (Kamvar et al., 2015).

### Sequencing data analysis

We used Illumina WGS and the assembled genomes of *A. duranensis* V 14167 and *A. ipaënsis* K 30076 to analyze the genomic composition of selected IpaDur1 plants. First, this involved determining alleles that distinguish the *A. duranensis* V 14167 and *A. ipaënsis* K 30076 genomes by mapping fragmented reference genomes and Illumina data from both accessions to the reference genome of *A. ipaënsis* gnm1. Then, also via mapping to the reference genome of *A. ipaënsis*, the numbers of these contrasting alleles in the WGS data of the selected IpaDur1 genotypes were counted. A normalized allele count was calculated to compensate for biases in the mapping of sequence data.

Therefore, the *A. duranensis* (AA genome) and *A. ipaënsis* (BB genome) gnm1 and gnm2 genomes were split into 10 kb genome fragments. For *A. duranensis*, gnm1 this resulted in 109 400 10 kb fragments (1.08 Gb), while gnm2 had 109 269 fragments (1.08 Gb). For *A. ipaënsis*, gnm1 had 135 752 fragments (1.35 Gb), and gnm2 had 143 871 fragments (1.43 Gb). In addition, WGS Illumina short reads, with coverage ranging from 75× to 110×, from two independent sources were used. The first, from Beijing Genomics Institute (BGI) (Shenzhen, China) consisted of 636 million reads for *A. duranensis* (approximately 160 Gb) and 644 million for *A. ipaënsis* (about 162 Gb). The second, from HudsonAlpha (Huntsville, Alabama) contributed 1.24 billion reads for *A. duranensis* (around 120 Gb) and 1.25 billion for *A. ipaënsis* (approximately 125 Gb). After quality filtering with BBMap (version 39.01) (Bushnell, 2014) to remove low‐quality reads, trim low‐quality bases, and cut adapter sequences, BGI reads were reduced to 621 million for *A. duranensis* (139 Gb) and *A. ipaënsis* (142 Gb), while HudsonAlpha reads were filtered down to 1.2 billion for both species (113 Gb for *A. duranensis* and 119 Gb for *A. ipaënsis*).

The filtered reads were then aligned to the *A. ipaënsis* reference genome gnm1 using BWA (version 0.7.17‐r1188) (Li & Durbin, 2009). Genetic variant calling was then performed on the alignments using BCFtools (version 1.15.1) (Danecek et al., 2021), followed by filtering to identify target AB variant positions. Variants were filtered to identify target AB variant positions based on four conditions: (1) The 10 kb genome fragments and whole‐genome Illumina short reads from both subgenomes (A and B) had to agree on a consensus subgenome allele. (2) Consensus alleles from WGS reads needed to account for at least 95% of the mapped reads at each variant position. (3) The 10 kb genome fragments required a minimum depth of 1. (4) Target AB variant positions were identified only where the consensus alleles differed between the A and B subgenomes.

Illumina WGS from the IpaDur1 plants, ID1.10.7.2.4.3.2 and ID1.10.13.5.4.15.5 from G1, and ID1.17.3.11.4.2.1 from G2, were then aligned to the *A. ipaënsis* reference genome gnm1 to call genetic variants at the identified target AB positions. Sequencing metrics indicated high‐quality data. Raw read counts ranged from 542 million to 617 million, providing substantial coverage across samples. Base error rates were consistently low at 0.03%, and GC content ranged from 36.24 to 37.35%, indicating typical genome characteristics.

Genetic variants were identified at the target AB variant positions, and raw AB allele counts were calculated for each sample. To normalize these raw counts, synthetic tetraploid peanut reads were generated by combining HudsonAlpha WGS reads in a 1:1 ratio of A and B subgenomes, based on genome sizes of 1.25 Gb for the A genome and 1.56 Gb for the B genome. The synthetic reads comprised 1.23 billion raw reads, totaling approximately 309.1 Gb. After filtering, the read count was reduced to 1.20 billion, corresponding to around 271.3 Gb. These reads were mapped onto the reference genome, and genetic variants were again identified at the target AB positions. Subgenome‐specific allele counts were then determined for the 1:1 synthetic read set. Sample normalization was performed using the following formulas:

$$\text{sample normalized}\;A=\frac{\left(\frac{\text{sample}\;A\;\text{count}}{\text{sample mean coverage}}\right)}{\left(\frac{\mathrm{norm}\;\mathrm{ref}\;A\;\text{count}}{\mathrm{norm}\;\mathrm{ref}\;\text{mean coverage}}\right)}$$

$$\text{sample normalized}\;B=\frac{\left(\frac{\text{sample}\;B\;\text{count}}{\text{sample mean coverage}}\right)}{\left(\frac{\mathrm{norm}\;\mathrm{ref}\;B\;\text{count}}{\mathrm{norm}\;\mathrm{ref}\;\text{mean coverage}}\right)}$$

Normalized allele counts for the IpaDur1 plants were used to create plots, visualizing allele distributions across chromosomes. These plots aimed to explore the genomic composition of the IpaDur1 plants and to compare the results with the genotypic data analysis results, which were summarized for this purpose using the “chromoMap” package (Anand & Rodriguez Lopez, 2022) in RStudio. The normalized density of allele count plots was constructed using “ggplot2.”

### Phenotypic data collection and analysis

Individual plants of IpaDur1, *A. duranensis* and *A. ipaënsis*, underwent phenotyping for multiple traits over the course of 6 years. These traits were flower color, flower color transitions, number of seeds, seed weight, total seed weight, maximum seed weight, number of pods, total pod weight, chlorophyll content as well as specific pod characteristics such as pod area, pod area convex hull, pod perimeter, pod mean radius, pod minimum radius, pod maximum radius, pod standard deviation radius, pod radius ratio, pod mean diameter, pod minimum diameter, pod maximum diameter, pod major axis, pod minor axis, pod eccentricity, pod solidity and pod circularity (A summary of all traits recorded each year is presented in Tables S6–S9) (Olivoto, 2022).

Photographs were taken of selected ID1.17 progenies, to document their white and variegated flower colors, as well as flower color transitions over time. Chlorophyll content was assessed using the SPAD 502 Chlorophyll Meter (Spectrum Technologies, Aurora, IL, USA) during the fourth and sixth years. Genotypes were evaluated twice per season with five replications: four from leaves near each pot corner and one from the main stem.

Pod traits were obtained by capturing pictures of pods against a dark background using a Canon EOS Rebel T3 camera with a Canon Macro Lens EF‐S 60 mm (Canon Inc., Tokyo, Japan). In the photographic documentation of peanut pods, a lateral perspective was adopted, capturing the pod's profile whenever possible. Consequently, the residual peg attachment typically appeared in one of the four corners. The acquired images were analyzed using “pliman” (Olivoto, 2022) in Rstudio. Initially, images of pods were adjusted in Paint 3D (Microsoft, Redmond, WA, USA) to remove any unwanted objects, and then imported into Rstudio. Pod characteristics were extracted using the analyze_objects() function. Subsequently, all pod measurements were calibrated to the image's known dpi, determined beforehand using the dpi() function on a ruler within the image, via the get_measures() function.

Phenotypic data analyses were conducted in RStudio. Traits were assessed for normality (Shapiro–Wilk test) and homoscedasticity (Levene's test) using functions from the “stats” and “car” packages (Fox & Weisberg, 2018). When assumptions were met, ANOVA followed by Tukey's post‐hoc test was performed with the “agricolae” package (de Mendiburu, 2023). If assumptions were violated, non‐parametric tests, such as Mann–Whitney *U* or Kruskal–Wallis, were applied using the “stats” package, with Dunn's test for multiple comparisons from the “FSA” package (Ogle et al., 2025) following the Kruskal–Wallis test. If the assumptions of normality or equal variance were not met, results were either reported based on *P*‐values or displayed using a compact letter display from pairwise comparisons with the “rcompanion” package (Mangiafico, 2025).

PCA, utilizing “adegenet,” “ade4,” “FactoMineR” and “factoextra,” was conducted on traits with numerical data, including number of seeds, seed weight, total seed weight, maximum seed weight, number of pods, total pod weight, pod area, pod area convex hull, pod perimeter, pod mean radius, pod minimum radius, pod maximum radius, pod standard deviation radius, pod radius ratio, pod mean diameter, pod minimum diameter, pod maximum diameter, pod major axis, pod minor axis, pod eccentricity, pod solidity, and pod circularity, on data collected from the third to the fifth year. Variables' contributions to the first five principal components were visualized using “corrplot” (Wei & Simko, 2017).

A tanglegram was generated to illustrate the relationship between genotypes and phenotypes. The tanglegram used “adegenet,” “ape,” and “phytools” (Revell, 2024), and “poppr.” To determine if selection affected seed weight and other related traits, multiple linear regressions were performed using “stats” to explore the relationships between seed weight, maximum seed weight, pod area, pod perimeter, and pod circularity, in relation to seed weight selection. Other charts for data visualization were generated with “data.tree” (Glur, 2023), “ggplot2,” “htmlwidgets” (Vaidyanathan et al., 2023), “networkD3” (Allaire, 2025), and “webshot” (Chang, 2023).

## AUTHOR CONTRIBUTIONS

The project was conceptualized by DJB and SCML‐B. SL was responsible for the collection of phenotypic data and conducted the analysis and visualization of both phenotypic and genotypic data, as well as the visualization of sequencing data. Sequencing data analysis and visualization were performed by BA and DJB. The manuscript was written by SL and DJB. The manuscript was approved by all authors.

## CONFLICT OF INTEREST

The authors declare no conflict of interest.

## Supporting information


**Figure S1.** Lineage tree of *Arachis duranensis* genotypes over the six years of advancement in the greenhouse.
**Figure S2.** Lineage tree of *Arachis ipaënsis* genotypes over the six years of advancement in the greenhouse.
**Figure S3.** IpaDur1 lineages exhibited superior responsiveness to selection compared with its wild parents over three years.
**Figure S4.** Maximum seed weight variation across genotypes and selections over three years.
**Figure S5.** Pod area, perimeter, and circularity variation across genotypes and seed weight selections.
**Figure S6.** Unrooted neighbor‐joining phylogenetic tree with 1000 bootstrap replicates of IpaDur1 lineages.
**Figure S7.** Flower color diversity and variability in IpaDur1 lineages.
**Figure S8.** Contributions and correlations of phenotypic variables in IpaDur1 and wild parents.
**Figure S9.** Contribution of phenotypic variables to the first five dimensions of principal component analysis.
**Figure S10.** Seed weight variation and seed yield relationship across IpaDur1 groups.

**Table S1.** Trait summary and statistical tests for seed and pod characteristics in wild diploid *Arachis* parents and IpaDur1 lineages.
**Table S2.** Regression analysis of seed and pod traits in wild diploid *Arachis* parents and IpaDur1 lineages.
**Table S3.** Final color‐coded matrix.
**Table S4.** Raw genotyping calls in wild diploid *Arachis* parents.
**Table S5.** Raw genotyping calls in DNA mixtures and tetraploid IpaDur1 lineages.
**Table S6.** Overview of collected phenotypic data in wild diploid *Arachis* parents and IpaDur1 lineages.
**Table S7.** Pod and seed traits of wild diploid *Arachis* parents and IpaDur1 lineages.
**Table S8.** Chlorophyll content of wild diploid *Arachis* parents and IpaDur1 lineages.
**Table S9.** Pod shape traits of wild diploid *Arachis* parents and IpaDur1 lineages.

## Data Availability

WGS data can be accessed in NCBI under BioProject accession PRJNA1238952. Sequencing analysis scripts are available at https://github.com/brianabernathy/ABGD. All other data generated or analyzed during this study are included in the supplementary files.

## References

[tpj70618-bib-0001] Akagi, T. , Jung, K. , Masuda, K. & Shimizu, K.K. (2022) Polyploidy before and after domestication of crop species. Current Opinion in Plant Biology, 69, 102255. Available from: 10.1016/j.pbi.2022.102255 35870416

[tpj70618-bib-0002] Allaire, J.J. (2025) Package ‘networkD3’ . Available at: https://cran.r‐project.org/package=networkD3

[tpj70618-bib-0003] Anand, L. & Rodriguez Lopez, C.M. (2022) ChromoMap: an R package for interactive visualization of multi‐omics data and annotation of chromosomes. BMC Bioinformatics, 23(1), 33. Available from: 10.1186/s12859-021-04556-z 35016614 PMC8753883

[tpj70618-bib-0004] Bertioli, D.J. , Abernathy, B. , Seijo, G. , Clevenger, J. & Cannon, S.B. (2020) Evaluating two different models of peanut's origin. Nature Genetics, 52(6), 557–559. Available from: 10.1038/s41588-020-0626-1 32393860

[tpj70618-bib-0005] Bertioli, D.J. , Cannon, S.B. , Froenicke, L. , Huang, G. , Farmer, A.D. , Cannon, E.K.S. et al. (2016) The genome sequences of *Arachis duranensis* and *Arachis ipaensis*, the diploid ancestors of cultivated peanut. Nature Genetics, 48(4), 438–446. Available from: 10.1038/ng.3517 26901068

[tpj70618-bib-0006] Bertioli, D.J. , Jenkins, J. , Clevenger, J. , Dudchenko, O. , Gao, D. , Seijo, G. et al. (2019) The genome sequence of segmental allotetraploid peanut *Arachis hypogaea* . Nature Genetics, 51(5), 877–884. Available from: 10.1038/s41588-019-0405-z 31043755

[tpj70618-bib-0007] Bertioli, D.J. , Seijo, G. , Freitas, F.O. , Valls, J.F.M. , Leal‐Bertioli, S.C.M. & Moretzsohn, M.C. (2011) An overview of peanut and its wild relatives. Plant Genetic Resources, 9(1), 134–149. Available from: 10.1017/S1479262110000444

[tpj70618-bib-0008] Bushnell, B. (2014) BBMap: a fast, accurate, splice‐aware aligner' .

[tpj70618-bib-0009] Chang, W. (2023) Package ‘webshot’ . Available at: https://cran.r‐project.org/package=webshot

[tpj70618-bib-0010] Clevenger, J. , Chu, Y. , Chavarro, C. , Agarwal, G. , Bertioli, D.J. , Leal‐Bertioli, S.C.M. et al. (2017) Genome‐wide SNP genotyping resolves signatures of selection and tetrasomic recombination in peanut. Molecular Plant, 10(2), 309–322. Available from: 10.1016/j.molp.2016.11.015 27993622 PMC5315502

[tpj70618-bib-0011] Comai, L. (2005) The advantages and disadvantages of being polyploid. Nature Reviews Genetics, 6(11), 836–846. Available from: 10.1038/nrg1711 16304599

[tpj70618-bib-0012] Danecek, P. , Bonfield, J.K. , Liddle, J. , Marshall, J. , Ohan, V. , Pollard, M.O. et al. (2021) Twelve years of SAMtools and BCFtools. GigaScience, 10(2), 1–4. Available from: 10.1093/gigascience/giab008 PMC7931819 33590861

[tpj70618-bib-0013] de Blas, F.J. , Leal‐Bertioli, S.C.M. , Seijo, J.G. , Abernathy, B.L. , Vaughn, J. , Ramachandran, D. et al. (2025) A single hybrid origin of cultivated peanut. The Plant Journal. The first manuscript of this three‐part series. Available from: 10.1111/tpj.70619 PMC12737836 41442708

[tpj70618-bib-0014] de Mendiburu, F. (2023) Package ‘agricolae’ . Available at: https://cran.r‐project.org/package=agricolae

[tpj70618-bib-0015] do Nascimento, E.F.D.M. , do Nascimento, E.F.d.M.B. , dos Santos, B.V. , Marques, L.O.C. , Guimarães, P.M. , Brasileiro, A.C.M. et al. (2018) The genome structure of *Arachis hypogaea* (Linnaeus, 1753) and an induced *Arachis* allotetraploid revealed by molecular cytogenetics. Comparative Cytogenetics, 12(1), 111–140. Available from: 10.3897/CompCytogen.v12i1.20334 29675140 PMC5904367

[tpj70618-bib-0016] Dray, S. , Dufour, A.B. & Chessel, D. (2007) The ade4 package‐II: two‐table and K‐table methods. R News, 7(2), 47–52.

[tpj70618-bib-0017] Fávero, A.P. , Simpson, C.E. , Valls, J.F.M. & Vello, N.A. (2006) Study of the evolution of cultivated peanut through crossability studies among *Arachis ipaënsis*, *A. duranensis*, and *A. hypogaea* . Crop Science, 46(4), 1546–1552. Available from: 10.2135/cropsci2005.09-0331

[tpj70618-bib-0018] Foncéka, D. , Tossim, H.A. , Rivallan, R. , Vignes, H. , Faye, I. , Ndoye, O. et al. (2012) Fostered and left behind alleles in peanut: interspecific QTL mapping reveals footprints of domestication and useful natural variation for breeding. BMC Plant Biology, 12, 1–16. Available from: 10.1186/1471-2229-12-26 22340522 PMC3312858

[tpj70618-bib-0019] Fox, J. & Weisberg, S. (2018) An R companion to applied regression. Thousand Oaks: Sage Publications.

[tpj70618-bib-0020] Gaeta, R.T. & Pires, C.J. (2010) Homoeologous recombination in allopolyploids: the polyploid ratchet. New Phytologist, 186(1), 18–28. Available from: 10.1111/j.1469-8137.2009.03089.x 20002315

[tpj70618-bib-0021] Gillies, C.B. (1989) Fertility and chromosome pairing: recent studies in plants and animals. Boca Raton: CRC Press, Inc.

[tpj70618-bib-0022] Glur, C. (2023) Package ‘data.tree’ . Available at: https://cran.r‐project.org/package=data.tree

[tpj70618-bib-0023] Hill, A.B. (1965) The environment and disease: association or causation? Proceedings of the Royal Society of Medicine, 58(5), 295–300. Available from: 10.1177/003591576505800503 14283879 PMC1898525

[tpj70618-bib-0024] Hilu, K.W. (1993) Polyploidy and the evolution of domesticated plants. American Journal of Botany, 80(12), 1494–1499. Available from: 10.1002/j.1537-2197.1993.tb15395.x

[tpj70618-bib-0025] Hume, D. (1748) An enquiry concerning human understanding. Houston: A. Millar.

[tpj70618-bib-0026] Jansky, S.H. , Charkowski, A.O. , Douches, D.S. , Gusmini, G. , Richael, C. , Bethke, P.C. et al. (2016) Reinventing potato as a diploid inbred line‐based crop. Crop Science, 56(4), 1412–1422. Available from: 10.2135/cropsci2015.12.0740

[tpj70618-bib-0027] Jombart, T. & Ahmed, I. (2011) Adegenet 1.3‐1: new tools for the analysis of genome‐wide SNP data. Bioinformatics, 27(21), 3070–3071. Available from: 10.1093/bioinformatics/btr521 21926124 PMC3198581

[tpj70618-bib-0028] Kamvar, Z.N. , Brooks, J.C. & Grünwald, N.J. (2015) Novel R tools for analysis of genome‐wide population genetic data with emphasis on clonality. Frontiers in Genetics, 6, 208. Available from: 10.3389/fgene.2015.00208 26113860 PMC4462096

[tpj70618-bib-0029] Kassambara, A. & Mundt, F. (2020) Package ‘factoextra’ . Available at: https://cran.r‐project.org/package=factoextra

[tpj70618-bib-0030] Korani, W. , Clevenger, J.P. , Chu, Y. & Ozias‐Akins, P. (2019) Machine learning as an effective method for identifying true single nucleotide polymorphisms in polyploid plants. The Plant Genome, 12(1), 180023. Available from: 10.3835/plantgenome2018.05.0023 PMC12962348 30951095

[tpj70618-bib-0031] Krapovickas, A. , Gregory, W.C. , Williams, D.E. & Simpson, C.E. (2007) Taxonomy of the genus *Arachis* (Leguminosae). Bonplandia, 16, 7–205 Available from: http://www.jstor.org/stable/41941433

[tpj70618-bib-0032] Lamon, S. , Abernathy, B.L. , Leal‐Bertioli, S.C.M. & Bertioli, D.J. (2025) Surprisingly frequent chromosomal instability in cultivated peanut. The Plant Journal. The third manuscript of this three‐part series. Available from: 10.1111/tpj.70617 41443178

[tpj70618-bib-0033] Lê, S. , Josse, J. & Husson, F. (2008) FactoMineR: a package for multivariate analysis. Journal of Statistical Software, 25, 1–18. Available from: 10.18637/jss.v025.i01

[tpj70618-bib-0034] Leal‐Bertioli, S.C.M. , Bertioli, D.J. , Guimarães, P.M. , Pereira, T.D. , Galhardo, I. , Silva, J.P. et al. (2012) The effect of tetraploidization of wild *Arachis* on leaf morphology and other drought‐related traits. Environmental and Experimental Botany, 84, 17–24. Available from: 10.1016/j.envexpbot.2012.04.005

[tpj70618-bib-0035] Leal‐Bertioli, S.C.M. , Leal‐Bertioli, S. , Shirasawa, K. , Abernathy, B. , Moretzsohn, M. , Chavarro, C. et al. (2015) Tetrasomic recombination is surprisingly frequent in allotetraploid *Arachis* . Genetics, 199(4), 1093–1105. Available from: 10.1534/genetics.115.174607 25701284 PMC4391553

[tpj70618-bib-0036] Leal‐Bertioli, S.C.M. , Moretzsohn, M.C. , Santos, S.P. , Brasileiro, A.C.M. , Guimarães, P.M. , Bertioli, D.J. et al. (2017) Phenotypic effects of allotetraploidization of wild *Arachis* and their implications for peanut domestication. American Journal of Botany, 104(3), 379–388. Available from: 10.3732/ajb.1600402 28341626

[tpj70618-bib-0037] Leal‐Bertioli, S.C.M. , Nascimento, E.F.M.B. , Chavarro, M.C.F. , Custódio, A.R. , Hopkins, M.S. , Moretzsohn, M.C. et al. (2021) Spontaneous generation of diversity in *Arachis* neopolyploids (*Arachis ipaënsis* × *Arachis duranensis*)^4x^ replays the early stages of peanut evolution. G3: Genes, Genomes, Genetics, 11(11), jkab289. Available from: 10.1093/g3journal/jkab289 34510200 PMC8527490

[tpj70618-bib-0038] Li, H. & Durbin, R. (2009) Fast and accurate short read alignment with Burrows–Wheeler transform. Bioinformatics, 25(14), 1754–1760. Available from: 10.1093/bioinformatics/btp324 19451168 PMC2705234

[tpj70618-bib-0039] Mangiafico, S.S. (2025) Package ‘rcompanion’ . Available at: https://cran.r‐project.org/package=rcompanion/

[tpj70618-bib-0040] McClintock, B. (1984) The significance of responses of the genome to challenge. Science, 226(4676), 792–801. Available from: 10.1126/science.15739260 15739260

[tpj70618-bib-0041] Meyer, R.S. , Duval, A.E. & Jensen, H.R. (2012) Patterns and processes in crop domestication: an historical review and quantitative analysis of 203 global food crops. New Phytologist, 196(1), 29–48. Available from: 10.1111/j.1469-8137.2012.04253.x 22889076

[tpj70618-bib-0042] MPIC Potato Research Report . (2023) Montcalm Research Center . Available at: https://www.canr.msu.edu/potatobg/Publications/field‐trial‐publications [Accessed 18th June 2025)]

[tpj70618-bib-0043] Ogle, D.H. , Doll, J.C. , Wheeler, A.P. & Dinno, A. (2025) Package ‘FSA’ . Available at: https://cran.r‐project.org/package=FSA

[tpj70618-bib-0044] Olivoto, T. (2022) Lights, camera, pliman! An R package for plant image analysis. Methods in Ecology and Evolution, 13(4), 789–798. Available from: 10.1111/2041-210X.13803

[tpj70618-bib-0045] Pandey, M.K. , Agarwal, G. , Kale, S.M. , Clevenger, J. , Nayak, S.N. , Sriswathi, M. et al. (2017) Development and evaluation of a high density genotyping “axiom‐*Arachis*” array with 58 K SNPs for accelerating genetics and breeding in groundnut. Scientific Reports, 7(1), 40577. Available from: 10.1038/srep40577 28091575 PMC5238394

[tpj70618-bib-0046] Paradis, E. & Schliep, K. (2019) Ape 5.0: an environment for modern phylogenetics and evolutionary analyses in R. Bioinformatics, 35(3), 526–528. Available from: 10.1093/bioinformatics/bty633 30016406

[tpj70618-bib-0047] R Core Team . (2023) R: a language and environment for statistical computing . Available at: https://www.r‐project.org/

[tpj70618-bib-0048] Renny‐Byfield, S. & Wendel, J.F. (2014) Doubling down on genomes: polyploidy and crop plants. American Journal of Botany, 101(10), 1711–1725. Available from: 10.3732/ajb.1400119 25090999

[tpj70618-bib-0049] Revell, L.J. (2024) Phytools 2.0: an updated R ecosystem for phylogenetic comparative methods (and other things). PeerJ, 12, e16505. Available from: 10.7717/peerj.16505 38192598 PMC10773453

[tpj70618-bib-0050] Salman‐Minkov, A. , Sabath, N. & Mayrose, I. (2016) Whole‐genome duplication as a key factor in crop domestication. Nature Plants, 2(8), 1–4. Available from: 10.1038/nplants.2016.115 27479829

[tpj70618-bib-0051] Samoluk, S.S. , Robledo, G. , Podio, M. , Chalup, L. , Ortiz, J.P.A. , Pessino, S.C. et al. (2015) First insight into divergence, representation and chromosome distribution of reverse transcriptase fragments from L1 retrotransposons in peanut and wild relative species. Genetica, 143, 113–125. Available from: 10.1007/s10709-015-9820-y 25633099

[tpj70618-bib-0052] Seijo, G. , Lavia, G.I. , Fernández, A. , Krapovickas, A. , Ducasse, D.A. , Bertioli, D.J. et al. (2007) Genomic relationships between the cultivated peanut (*Arachis hypogaea*, Leguminosae) and its close relatives revealed by double GISH. American Journal of Botany, 94(12), 1963–1971. Available from: 10.3732/ajb.94.12.1963 21636391

[tpj70618-bib-0053] Selmecki, A.M. , Maruvka, Y.E. , Richmond, P.A. , Guillet, M. , Shoresh, N. , Sorenson, A.L. et al. (2015) Polyploidy can drive rapid adaptation in yeast. Nature, 519(7543), 349–351. Available from: 10.1038/nature14187 25731168 PMC4497379

[tpj70618-bib-0054] Soltis, D.E. , Visger, C.J. & Soltis, P.S. (2014) The polyploidy revolution then…And now: Stebbins revisited. American Journal of Botany, 101(7), 1057–1078. Available from: 10.3732/ajb.1400178 25049267

[tpj70618-bib-0055] Soltis, P.S. , Marchant, D.B. , van de Peer, Y. & Soltis, D.E. (2015) Polyploidy and genome evolution in plants. Current Opinion in Genetics and Development, 35, 119–125. Available from: 10.1016/j.gde.2015.11.003 26656231

[tpj70618-bib-0056] Soltis, P.S. & Soltis, D.E. (2012) Polyploidy and genome evolution. Berlin: Springer. Available from: 10.1007/978-3-642-31442-1

[tpj70618-bib-0057] Stalker, H.T. & Wilson, R.F. (2016) Peanuts: genetics, processing, and utilization. Amsterdam: Elsevier.

[tpj70618-bib-0058] Stebbins, G.L. (1971) Chromosomal evolution in higher plants. Reading: Addison‐Wesley.

[tpj70618-bib-0059] Tossim, H.A. , Nguepjop, J.R. , Diatta, C. , Sambou, A. , Seye, M. , Sane, D. et al. (2020) Assessment of 16 Peanut (*Arachis hypogaea* L.) CSSLs derived from an interspecific cross for yield and yield component traits: QTL validation. Agronomy, 10(4), 583. Available from: 10.3390/agronomy10040583

[tpj70618-bib-0060] Vaidyanathan, R. , Xie, Y. , Allaire, J.J. , Cheng, J. , Sievert, C. , Russell, K. et al. (2023) Package ‘htmlwidgets’ . Available at: https://cran.r‐project.org/package=htmlwidgets

[tpj70618-bib-0061] Voorrips, R.E. (2025) Package ‘fitPoly’ . Available at: https://cran.r‐project.org/package=fitPoly

[tpj70618-bib-0062] Voorrips, R.E. , Gort, G. & Vosman, B. (2011) Genotype calling in tetraploid species from bi‐allelic marker data using mixture models. BMC Bioinformatics, 12, 1–11. Available from: 10.1186/1471-2105-12-172 21595880 PMC3121645

[tpj70618-bib-0063] Wang, T. , van Dijk, A.D.J. , Zhao, R. , Bonnema, G. & Wang, X. (2024) Contribution of homoeologous exchange to domestication of polyploid brassica. Genome Biology, 25(1), 231. Available from: 10.1186/s13059-024-03370-z 39192349 PMC11350971

[tpj70618-bib-0064] Wei, T. & Simko, V. (2017) R package “corrplot”: visualization of a correlation matrix . Available at: https://github.com/taiyun/corrplot

[tpj70618-bib-0065] Wickham, H. , Chang, W. , Henry, L. , Pedersen, T.L. , Takahashi, K. , Wilke, C. et al. (2025) Package ‘ggplot2’ . Available at: https://cran.r‐project.org/package=ggplot2

[tpj70618-bib-0066] Wood, T.E. , Takebayashi, N. , Barker, M.S. , Mayrose, I. , Greenspoon, P.B. & Rieseberg, L.H. (2009) The frequency of polyploid speciation in vascular plants. Proceedings of the National Academy of Sciences, 106(33), 13875–13879. Available from: 10.1073/pnas.0811575106 PMC2728988 19667210

