# Supplementary material for: From bottleneck to boom: Polyploidy, genetic instability and response to artificial selection resolve the peanut paradox
Source: Plant J. 2025 Dec 24;124(6):e70618. doi: 10.1111/tpj.70618 (PMC12737905; doi:10.1111/tpj.70618)
Supplement: Supplementary file 1 — Figure S1. Lineage tree of Arachis duranensis genotypes over the six years of advancement in the greenhouse. Figure S2. Lineage tree of Arachis ipaënsis genotypes over the six years of advancement in the greenhouse. Figure S3. IpaDur1 lineages exhibited superior responsiveness to selection compared with its wild parents over three years. Figure S4. Maximum seed weight variation across genotypes and selections over three years. Figure S5. Pod area, perimeter, and circularity variation across genotypes and seed weight selections. Figure S6. Unrooted neighbor‐joining phylogenetic tree with 1000 bootstrap replicates of IpaDur1 lineages. Figure S7. Flower color diversity and variability in IpaDur1 lineages. Figure S8. Contributions and correlations of phenotypic variables in IpaDur1 and wild parents. Figure S9. Contribution of phenotypic variables to the first five dimensions of principal component analysis. Figure S10. Seed weight variation and seed yield relationship across IpaDur1 groups. [file TPJ-124-0-s001.docx]

**Supplementary Figures for**

**From Bottleneck to Boom:**

**Polyploidy, Genetic Instability and Response to Artificial Selection**

**Resolve the Peanut Paradox**

Samuele Lamon^1^ ([Samuele.Lamon@uga.edu](mailto:Samuele.Lamon@uga.edu); ORCID: [0000-0002-8475-7785](https://orcid.org/0000-0002-8475-7785)), Brian Abernathy^2^ ([bla@uga.edu](mailto:bla@uga.edu)), Soraya C. M. Leal-Bertioli^1,2,3^ ([sbertioli@uga.edu](mailto:sbertioli@uga.edu); ORCID: [0000-0002-9683-5357](https://orcid.org/0000-0002-9683-5357)) and David J. Bertioli^1,2,4^ ([bertioli@uga.edu](mailto:bertioli@uga.edu); ORCID: [0000-0003-0294-7284](https://orcid.org/0000-0003-0294-7284))

^1^Institute of Plant Breeding, Genetics and Genomics, University of Georgia, Athens, GA 30602, USA;

^2^Center for Applied Genetic Technologies, University of Georgia, Athens, GA 30602, USA;

^3^Department of Plant Pathology, University of Georgia, Athens, GA 30602, USA;

^4^Department of Crop & Soil Sciences, University of Georgia, Athens, GA 30602, USA;

Corresponding author: David J. Bertioli.

**
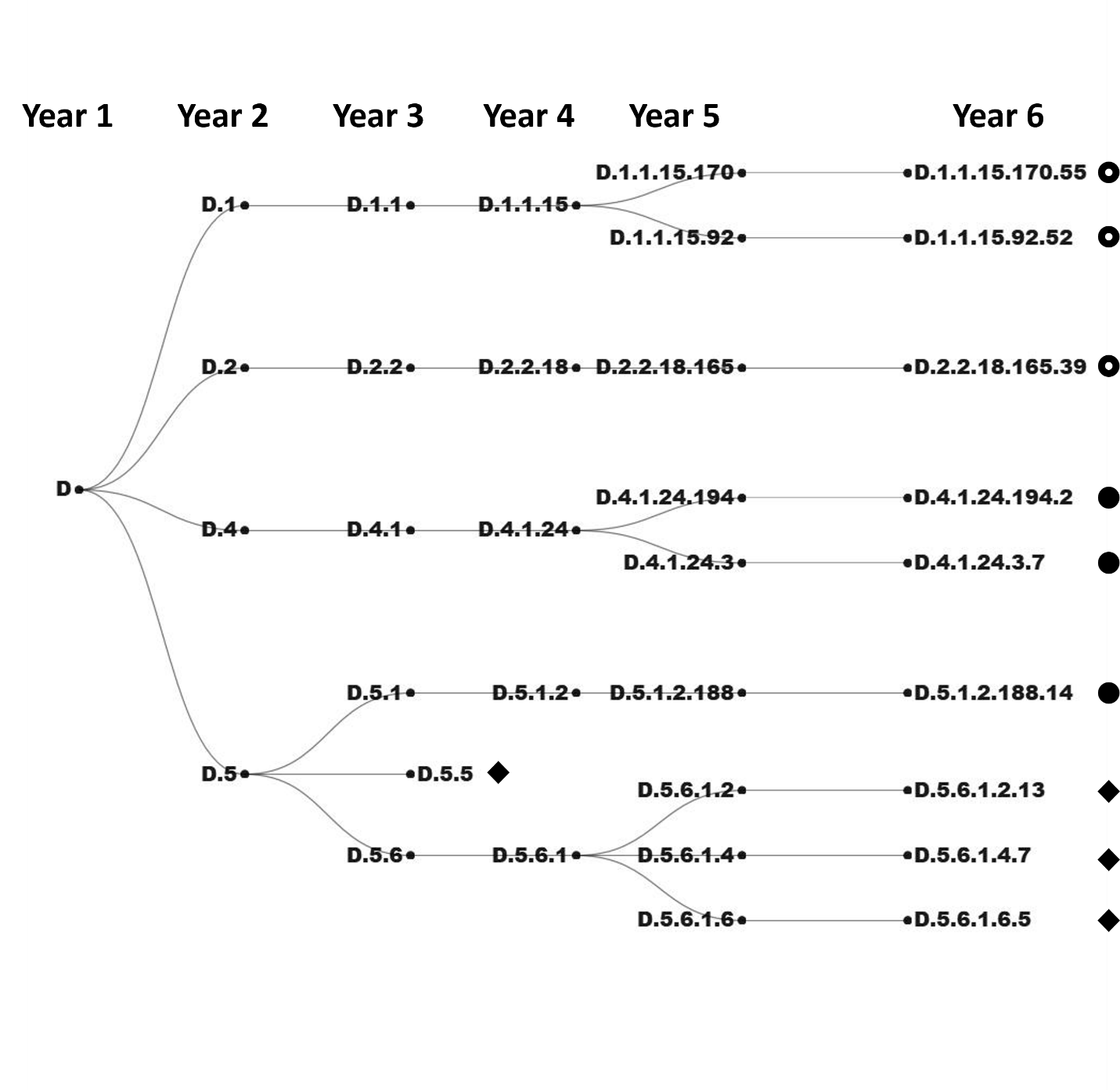
**

**Fig. S1:** **Lineage tree of *Arachis duranensis* genotypes over the six years of advancement in the greenhouse**. Only seed-producing genotypes are depicted, and all considered genotypes had yellow flowers. Circles indicate heavy seed weight selection; open circles indicate light seed weight selection, while rhombuses represent average seed weight selection.

**
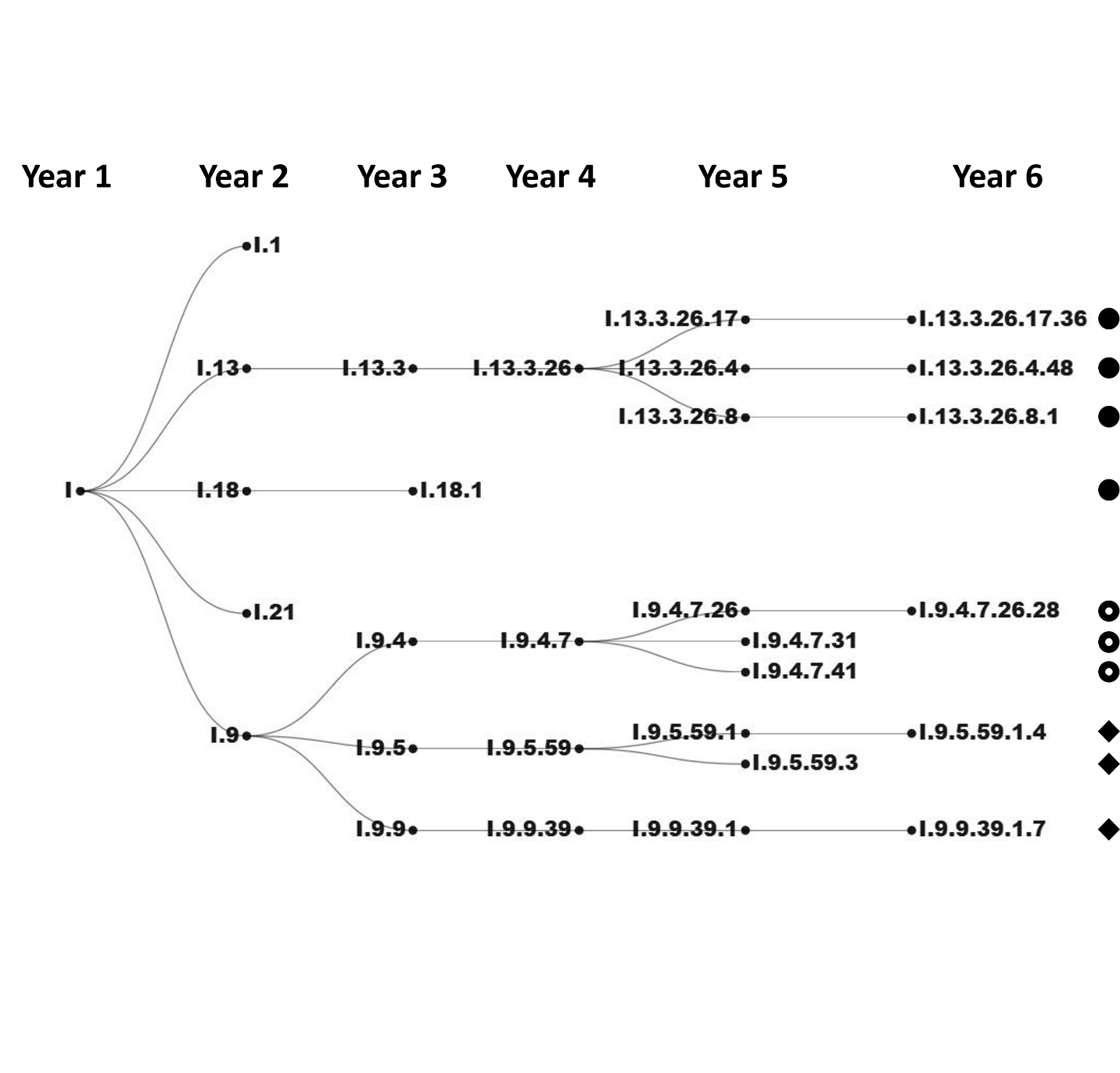
**

**Fig. S2:** **Lineage tree of *Arachis ipaënsis* genotypes over the six years of advancement in the greenhouse**. Only seed-producing genotypes are depicted, and all considered genotypes had orange flowers. Circles indicate heavy seed weight selection; open circles indicate light seed weight selection, while rhombuses represent average seed weight selection.


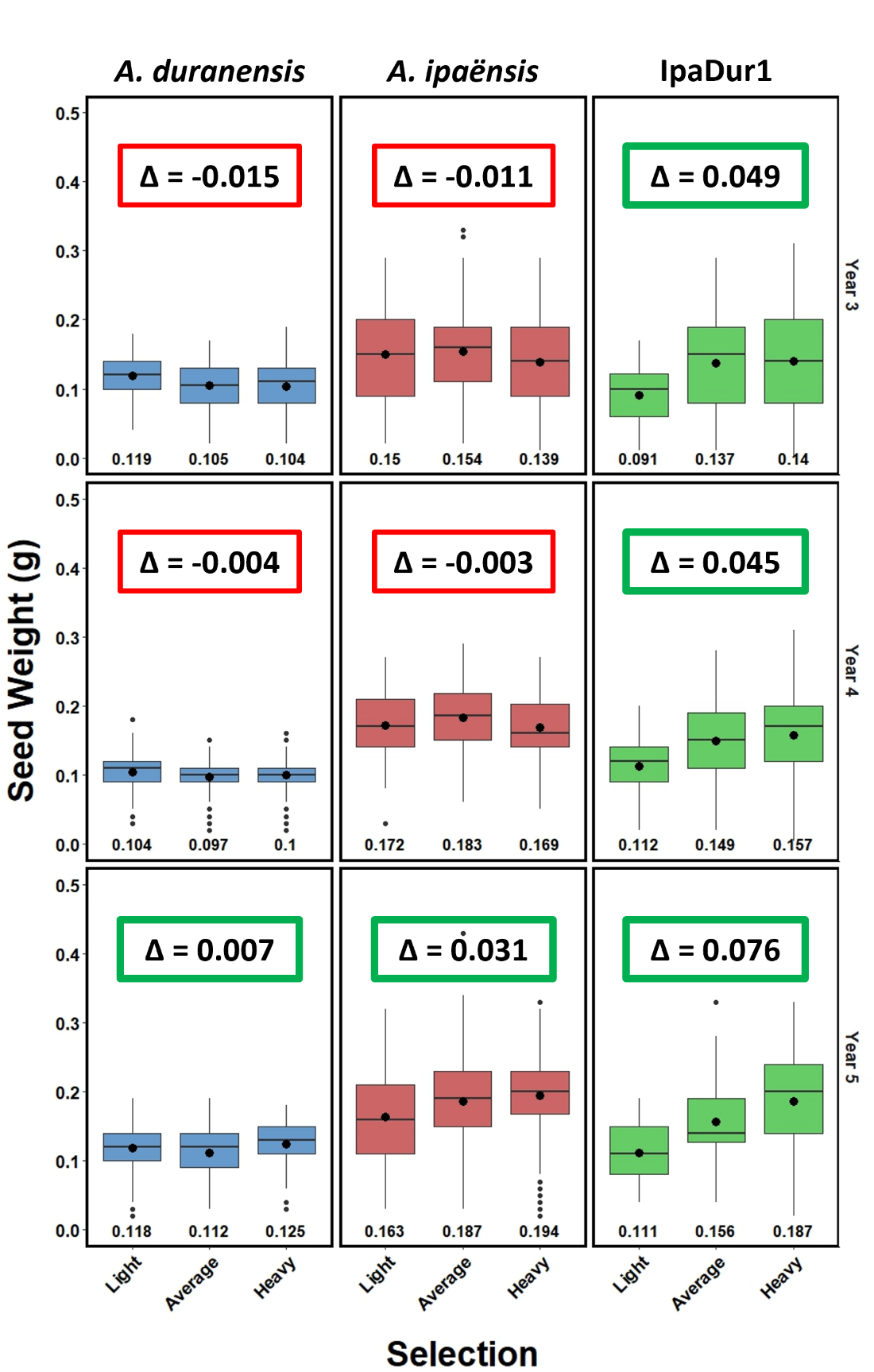


**Fig. S3:** **IpaDur1 lineages exhibited superior responsiveness to selection compared to its wild parents over three years**. Extremes in both light and heavy seed weights achieved weights comparable to those of *Arachis duranensis* and *A. ipaënsis*. The box plots display seed weight variations in three peanut genotypes: *A. duranensis* (blue), *A. ipaënsis* (red), and IpaDur1 lineages (green), selected for light, average, and heavy seed weights across three years of selection. Black dots and black numbers represent average seed weights for each genotype-selection combination. Additionally, Δ denotes the difference between the heavy and light seed weight selections. Highlighted in red are Δ showing ineffective selection, in green Δ showing effective selection.


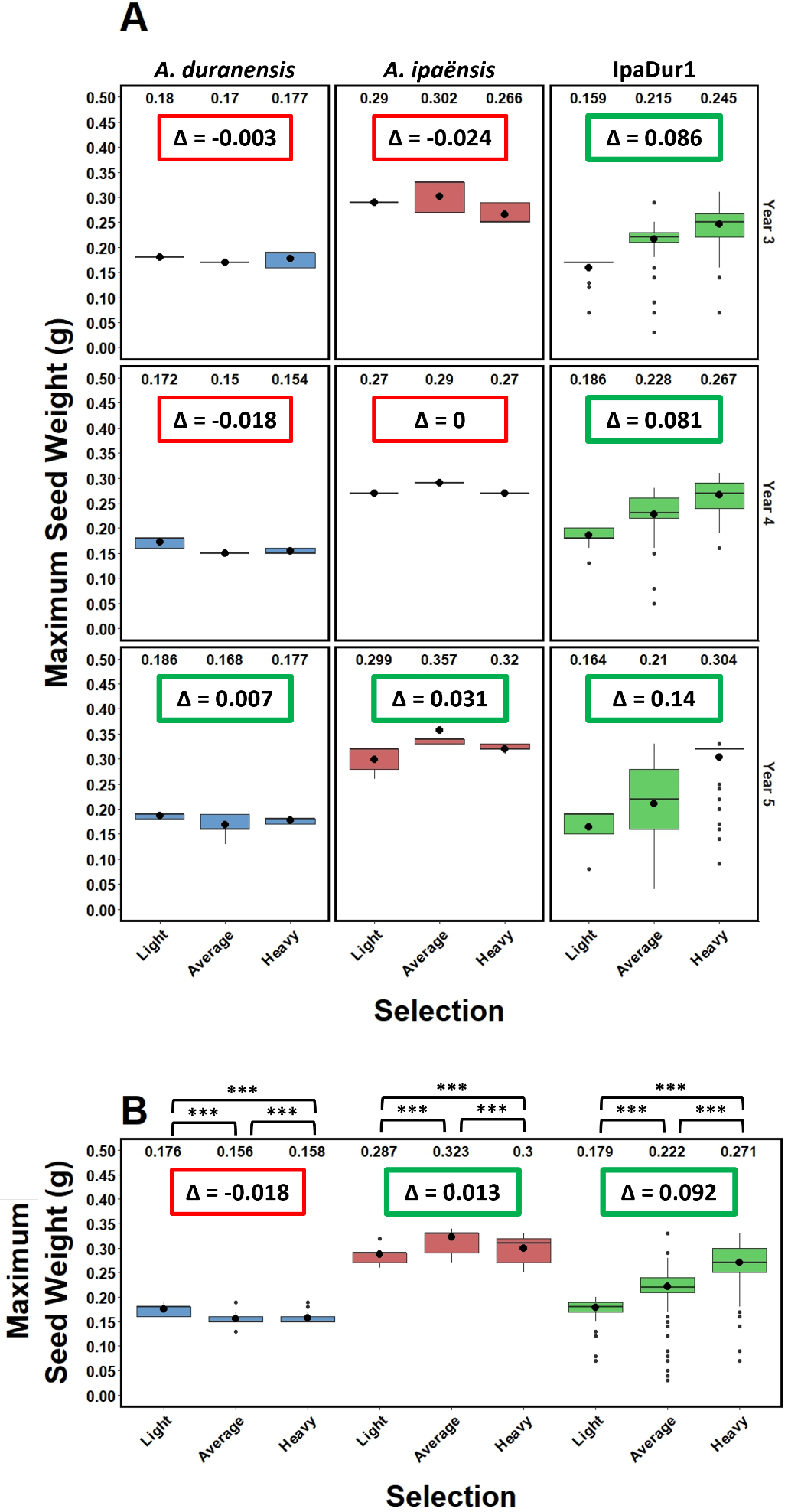


**Fig. S4:** **Maximum seed weight variation across genotypes and selections over three years**. Box plots by year representing maximum seed weight variations in three peanut genotypes: *Arachis duranensis* (blue), *A. ipaënsis* (red), and IpaDur1 lineages (green), selected for light, average, and heavy seed weights across three years of selection (panel A) and all years combined data (panel B). Black dots and black numbers represent average maximum seed weight for each genotype-selection combination. Δ denotes the difference between heavy and light maximum seed weight selections. Highlighted in red are Δ showing ineffective selection, in green Δ showing effective selection. Statistical significance of combined data was evaluated using the Kruskal-Wallis non-parametric rank sum test followed by Dunn’s post hoc test (***p < 0.001).

**
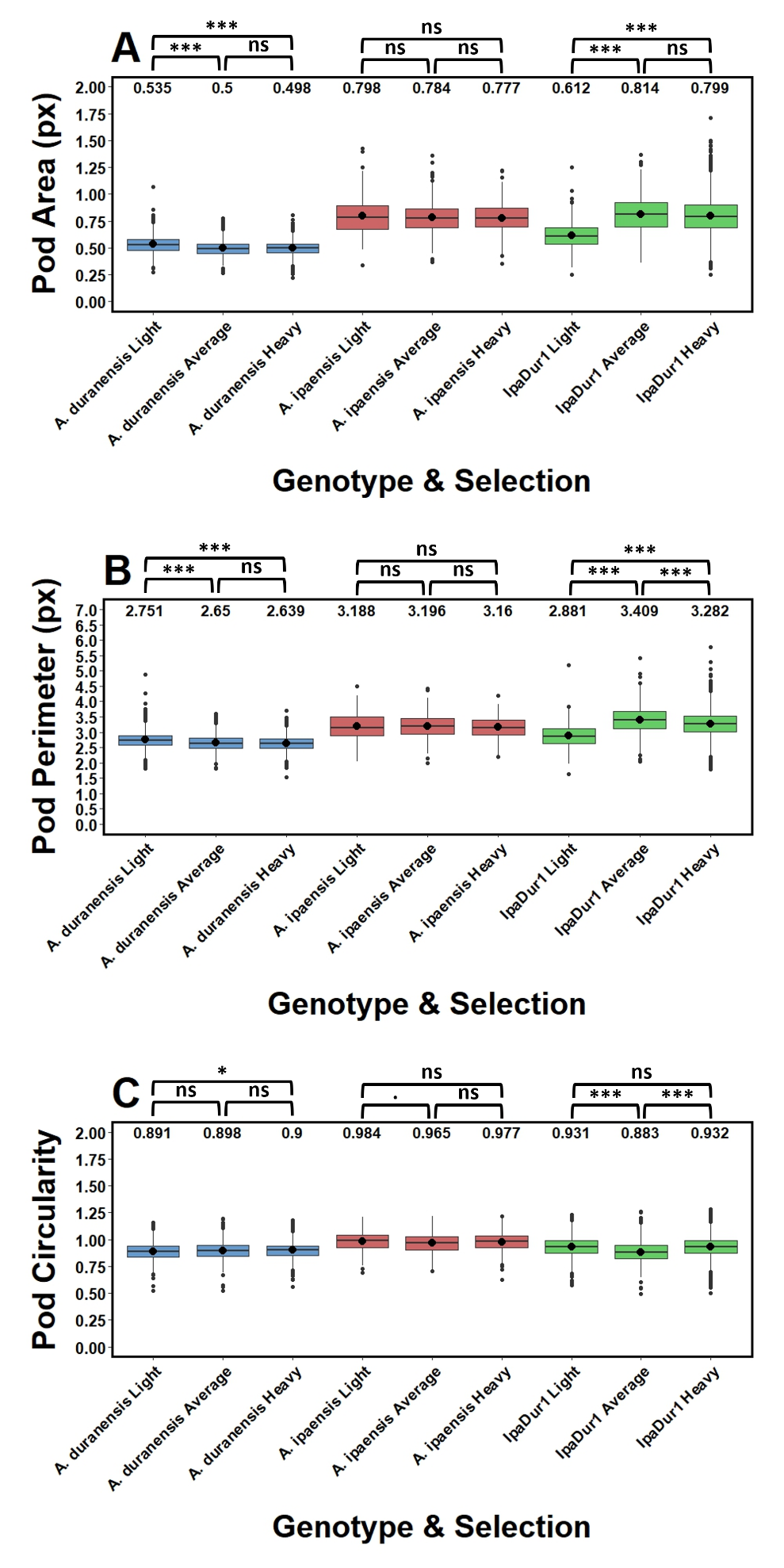
**

**Fig. S5:** **Pod area, perimeter, and circularity variation across genotypes and seed weight selections**. Box plots representing pod area (panel A), pod perimeter (panel B), and pod circularity (panel C) variations in three peanut genotypes: *Arachis duranensis* (blue), *A. ipaënsis* (red), and IpaDur1 lineages (green), selected for light, average, and heavy seed weights. Black dots and black numbers represent average pod area, pod perimeter, and pod circularity for each genotype-selection combination. Statistical significance was assessed using Kruskal-Wallis non-parametric rank sum test and Dunn’s post hoc test (.p < 0.1, *p < 0.05, **p < 0.01, ***p < 0.001). "ns" denotes non-significant differences.

**
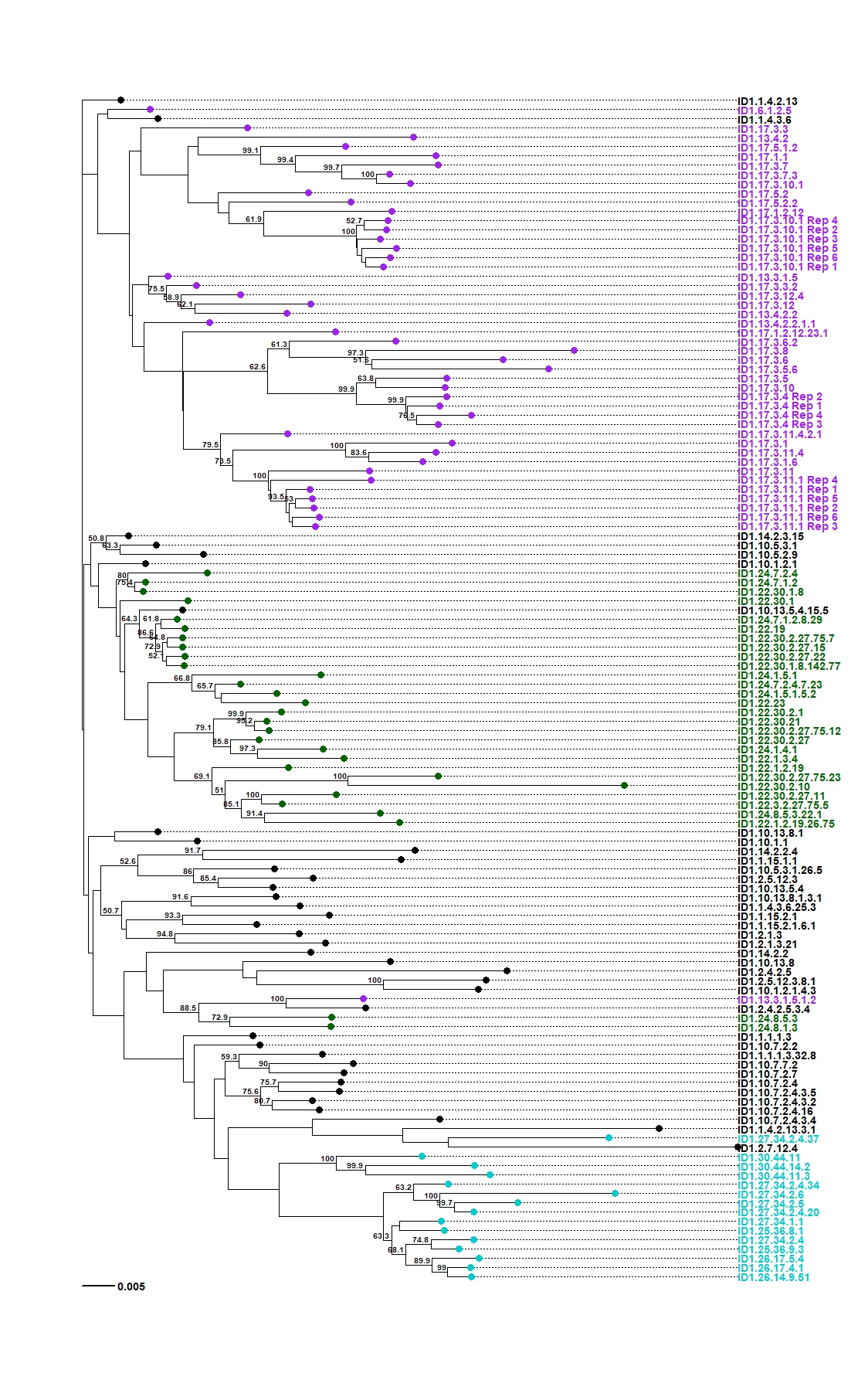
**

**Fig. S6:** **Unrooted neighbor-joining phylogenetic tree with 1000 bootstrap replicates of IpaDur1 lineages**. G1 (black), G2 (purple), G3 (green), and G4 (cyan) are well separated.


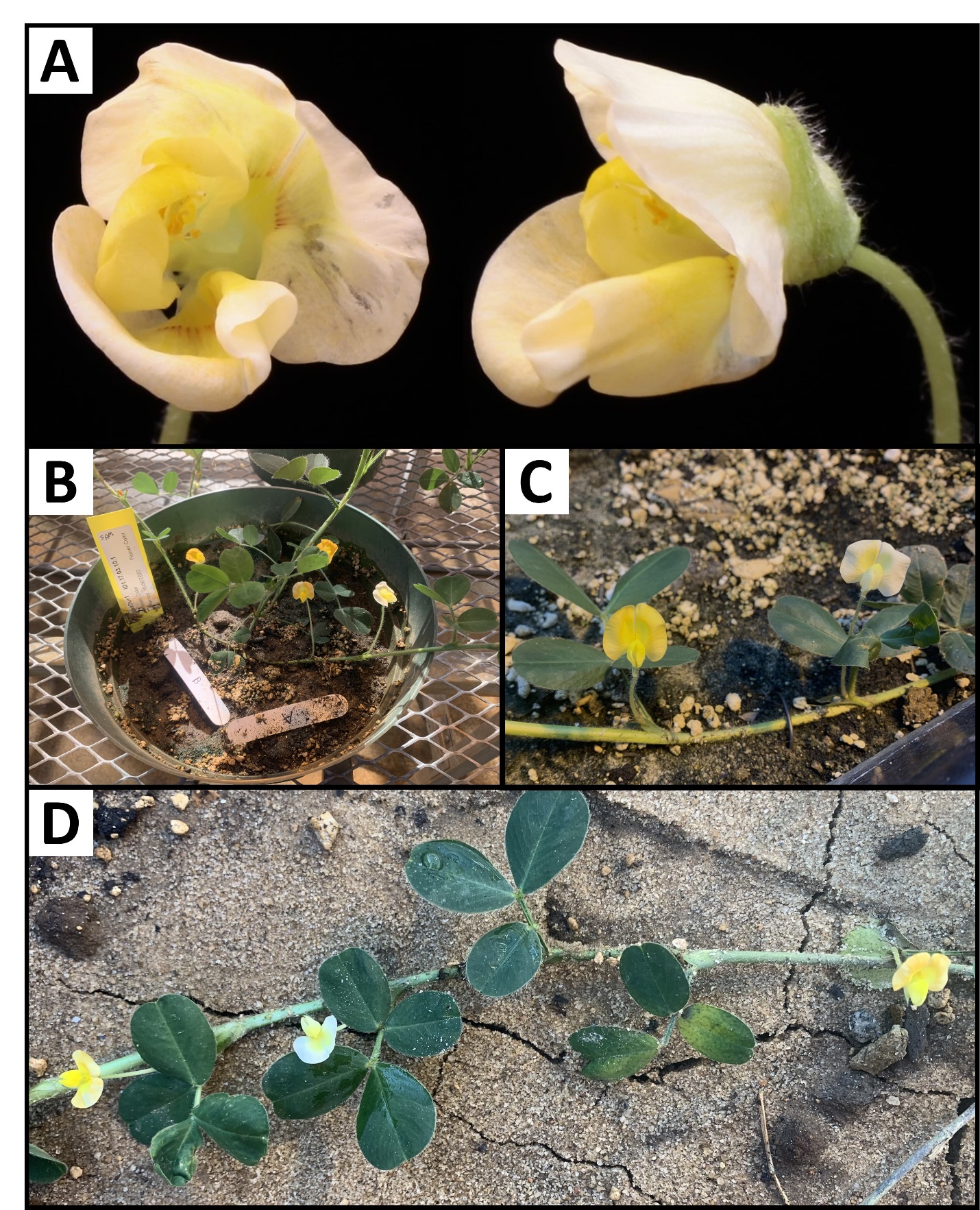


**Fig. S7:** **Flower color diversity and variability in IpaDur1 lineages**. Panel A shows ID1.17.3.12.4, featuring a distinctive white flower with a modified shape, viewed from the front (left) and the left side (right). IpaDur1 lineages can exhibit either a uniform flower color throughout the entire plant or diverse flower colors (B, ID1.17.3.10.1; C, ID1.17.3.11.1; D, ID1.17.3.8.1) within the same plant, where individual branches may bear flowers of varying colors and patterns.


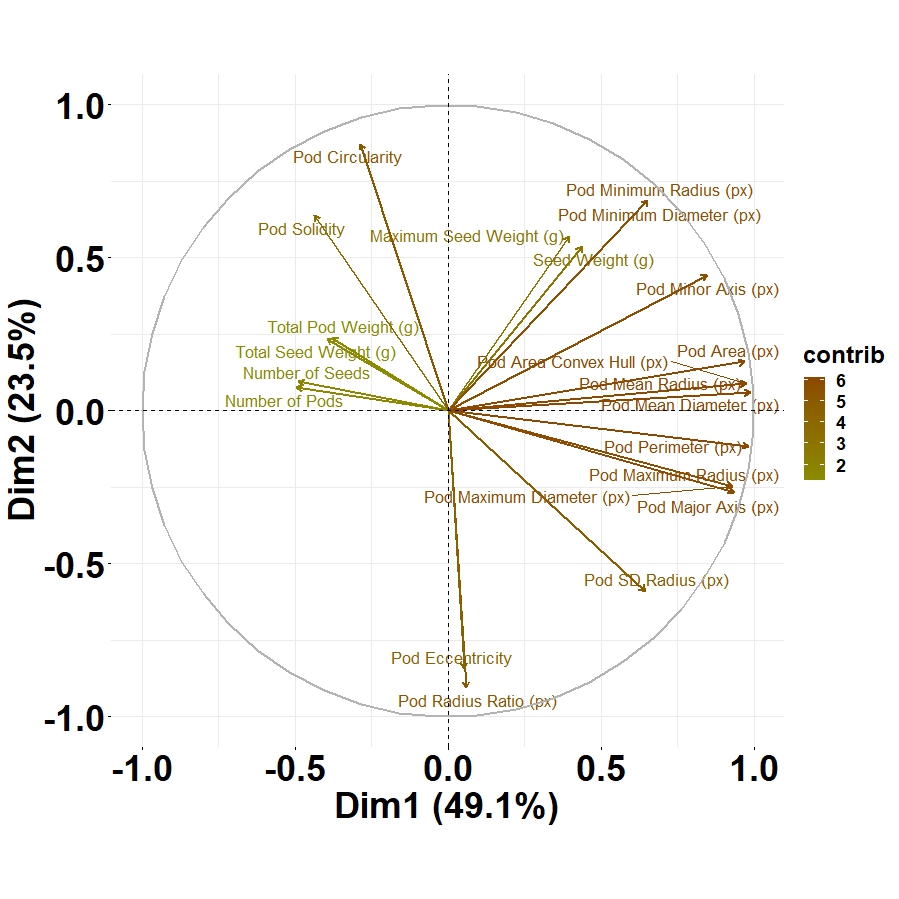


**Fig. S8:** **Contributions and correlations of phenotypic variables in IpaDur1 and wild parents**. Phenotypic variables related to pod size are the ones that mainly contribute to the principal component analysis variability.


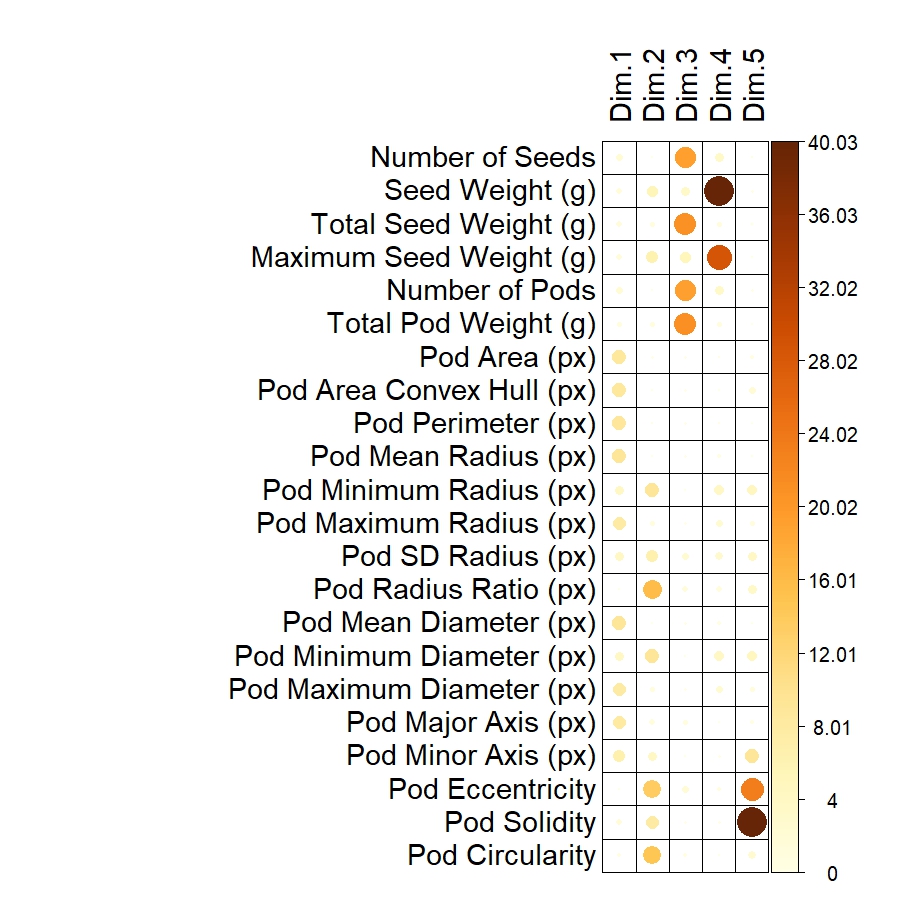


**Fig. S9:** **Contribution of phenotypic variables to the first five dimensions of principal component analysis.**


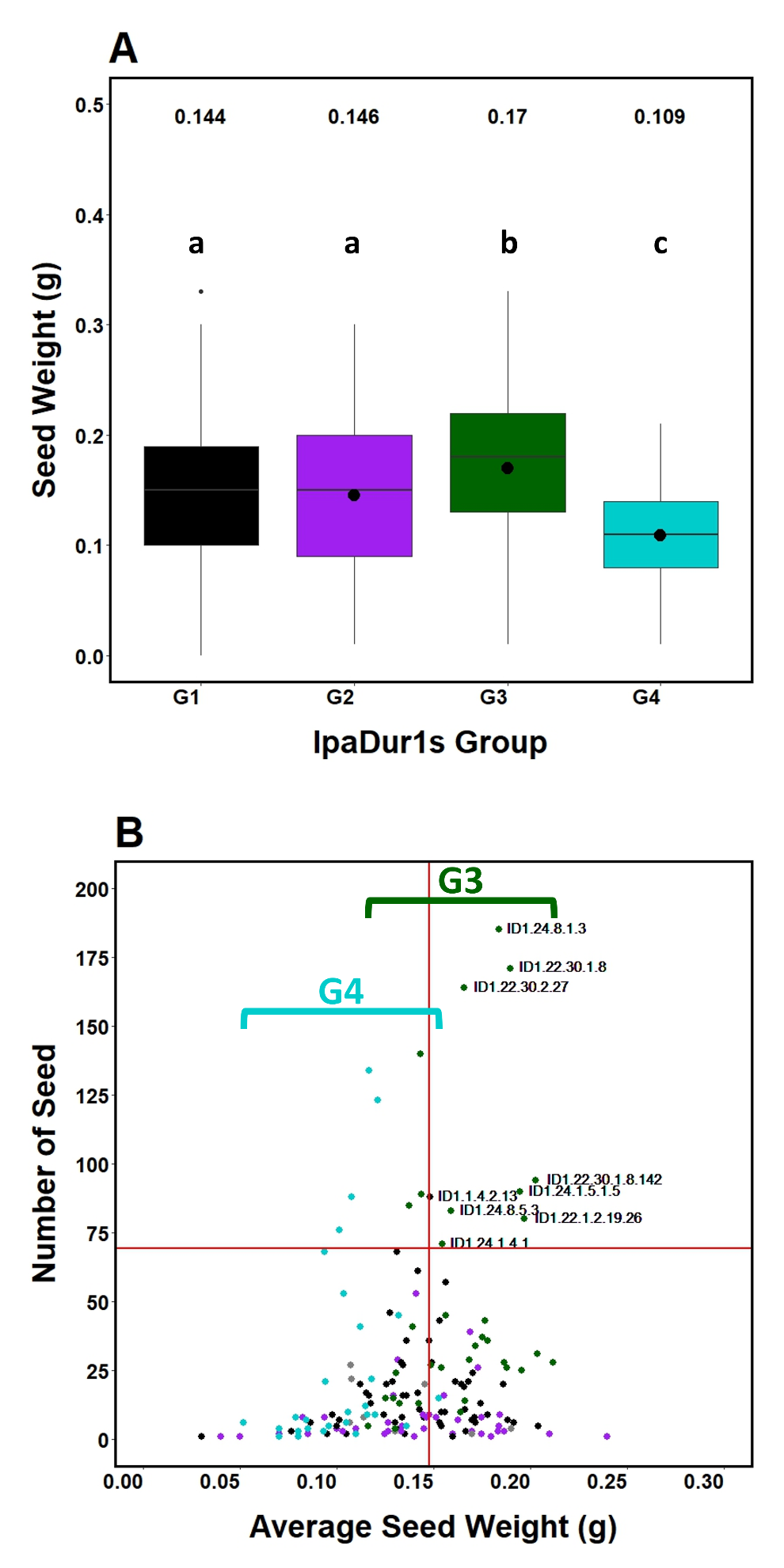


**Fig. S10:** **Seed weight variation and seed yield relationship across IpaDur1 groups**. Panel A displays box plots illustrating the variation in seed weight among IpaDur1 lineages, categorized into groups. Levene’s test, Shapiro-Wilk test, and Kruskal-Wallis test all yielded p-values < 0.001, indicating significant differences among groups. Post-hoc grouping separation was performed using Dunn’s post hoc test. In panel B, a scatter plot showcases the relationship between the number of seeds and the average seed weight for IpaDur1 genotypes across five years. Genotypes exceeding the average values for both number of seeds and average seed weight are denoted by black labels (highlighted by red lines). Notably, eight out of nine high-yield IpaDur1 lineages are exclusively progenies of ID1.22 and ID1.24, originating from either the fourth or fifth year. The color scheme indicates the grouping: G1 (black), G2 (purple), G3 (green), and G4 (cyan).
